# KDM1A promotes thyroid cancer progression and maintains stemness through the Wnt/β-catenin signaling pathway

**DOI:** 10.7150/thno.66142

**Published:** 2022-01-03

**Authors:** Wei Zhang, Xianhui Ruan, Yaoshuang Li, Jingtai Zhi, Linfei Hu, Xiukun Hou, Xianle Shi, Xin Wang, Jinpeng Wang, Weike Ma, Pengfei Gu, Xiangqian Zheng, Ming Gao

**Affiliations:** 1 Department of Thyroid and Neck Tumor, Tianjin Medical University Cancer Institute and Hospital, National Clinical Research Center for Cancer, Key Laboratory of Cancer Prevention and Therapy, Tianjin's Clinical Research Center for Cancer, Tianjin 300060, China; 2 Department of Breast and Thyroid Surgery, Tianjin Union Medical Center, No.190 Jieyuan Road, Hongqiao District, Tianjin 300121, P R China; 3 School of Medicine, Nankai University, Tianjin 300071, China; 4 Department of Endocrinology, Health Management Center, Tianjin Union Medical Center, Nankai University Affiliated Hospital, 190 of Jieyuan Road, Hongqiao District, Tianjin, 300121, China; 5 Department of Otolaryngology-Head and Neck Surgery, Tianjin First Center Hospital, Nankai District of Tianjin, Institute of Otolaryngology of Tianjin, Key Laboratory of Auditory Speech and Balance Medicine, Key Clinical Discipline of Tianjin (Otolaryngology), Otolaryngology Clinical Quality Control Centre, Rehabilitation Road No. 24, Tianjin, 300192, China; 6 Department of Medicine, Columbia Center for Human Development, Columbia University Irving Medical Center, New York, NY 10032, USA; 7 Department of Pathology, Tianjin Medical University Cancer Institute and Hospital, National Clinical Research Center for Cancer, Key Laboratory of Cancer Prevention and Therapy, Tianjin's Clinical Research Center for Cancer, Tianjin Medical University, 300060, Tianjin, China

**Keywords:** KDMIA, thyroid cancer, Wnt/β-catenin pathway, stemness, target therapy

## Abstract

**Background:** Cancer stem cells (CSCs) are highly tumorigenic, chemotherapy-resistant, tumor growth-sustaining, and are implicated in tumor recurrence. Previous studies have shown that lysine-specific histone demethylase 1A (KDM1A) is highly expressed in several human malignancies and CSCs. However, the role of KDM1A in CSCs and the therapeutic potential of KDM1A inhibitors for the treatment of the advanced thyroid cancer are poorly understood.

**Methods:** Firstly, KDM1A was identified as an important epigenetic modifier that maintained the stemness of thyroid cancer through a mini histone methylation modifier screen and confirmed in thyroid cancer tissues and cell lines. RNA sequence was performed to discover the downstream genes of KDM1A. The underlying mechanisms were further investigated by ChIP, IP and dual luciferase reporter assays, gain and loss of function assays.

**Results:** Here we report that KDM1A regulates the stemness of thyroid cancer and promotes thyroid cancer progression via the Wnt/β-catenin pathway. Mechanistically, KDM1A down-regulates two antagonists of the canonical Wnt pathway, APC2 and DKK1, by demethylating H3K4me1/2 of the APC2 promoter region and the nonhistone substrate HIF-1α, resulting in the inhibition of APC2 transcription and the activation of the HIF-1α/microRNA-146a/DKK1 axis. Importantly, we also demonstrate that GSK-LSD1, a highly selective inhibitor of KDM1A, significantly inhibits thyroid cancer progression and enhances the sensitivity of thyroid cancer to chemotherapy.

**Conclusions:** KDM1A plays an important role in thyroid cancer progression and maintains stemness, our study provides a new strategy for the therapy of advanced thyroid cancer.

## Introduction

Thyroid cancer is the most common endocrine malignant tumor, and its global incidence has been rising continuously in recent decades 1, 2. Follicular thyroid cell-derived tumors account for the vast majority of thyroid malignancies, including papillary thyroid carcinoma (PTC), follicular thyroid carcinoma (FTC), poorly differentiated thyroid carcinoma (PDTC) and anaplastic thyroid carcinoma (ATC) 3. PTC is a low-grade malignant tumor, and is the most common pathological type of thyroid cancer accounting for 80% ~ 85% of thyroid cancers in the population. These patients often have a good prognosis with conventional surgical treatment and radioactive iodine treatment 4, while 20%~30% of PTC patients experience recurrence, and 5% ~ 10% have progressive and refractory disease 5. ATC accounts for less than 2% of thyroid cancer, but it has an extremely poor prognosis and comprises more than 50% of thyroid-related mortalities 6. However, there are few treatment options available for patients with advanced disease, including radioiodine-resistant and metastatic differentiated thyroid cancer and ATC. Therefore, identifying new biomarkers and potential therapeutic targets for advanced thyroid cancer is of great significance.

Cancer stem cells (CSCs) are self-renewing cells capable of producing heterogeneous cancer cells and maintaining the vitality of the cancer cell population through self-renewal and infinite proliferation 7. CSCs greatly account for the survival, metastasis, proliferation and recurrence of cancer 8. Moreover, CSCs feature various drug-resistant genes and are not sensitive to conventional cancer treatment 9, 10. Previous studies have suggested that CSCs also profoundly influence the progression of thyroid cancer 11 and that the enrichment of CSCs in ATCs is much higher than that of PTCs 12. Thus, therapies targeting CSCs may become a cornerstone of advanced thyroid cancer therapy in the future.

Epigenetics is a principal branch of research on the pathogenesis of cancer and provides many alternative novel treatment strategies 13, 14. As one of the most well studied epigenetic modifications, histone methylation plays important roles in cancer initiation and development and has become the focus of pharmacological tumor interventions 15. Histone methylation is dynamically regulated by methyltransferases and demethylases. KDM1A (also known as LSD1) is the first discovered histone lysine demethylase, and has the ability to demethylase H3K4me1/2 and H3K9me1/2 at target loci in a context-dependent manner 16, 17. Generally, methylation on histone H3 lysine 4 is linked to transcriptional activation, while methylation of histone H3 at lysine 9 is considered as a transcriptional repression mark 18. KDM1A has already been proven to be involved in multiple biological processes of cancer progression, including proliferation 19, epithelial-mesenchymal transformation 20, 21, senescence 22, the maintenance of stem cell pluripotency 23, 24 and multidrug resistance 10. Nevertheless, its function in advanced thyroid cancer is scarcely understood.

In this study, we identified KDM1A as an important epigenetic modifier that maintains the stemness of thyroid cancer through a mini histone methylation modifier screen. Inhibition of KDM1A attenuates cancer stemness and promotes thyroid cancer chemosensitivity *in vitro* and *in vivo* via inhibiting the Wnt/β-catenin signaling pathway. Furthermore, pharmacologic inhibition of KDM1A suppresses thyroid cancer progression and enhances the antitumor effects of chemotherapy. Therefore, our study provides a promising treatment strategy for advanced thyroid cancer.

## Materials and Methods

### Cell lines and cell culture

Nthy-ori-3-1, KTC1, K1, TPC1, BCPAP, 8305C, 8505C, C643, CAL-62 and ACT1 cell lines were all purchased from American Type Culture Collection. All cell lines were identified by STR analysis. All cell lines were cultured in RPMI-1640 or DMEM (Gibco) supplemented with 10% fetal bovine serum and kept in a humid atmosphere of 5% CO_2_ at 37 °C.

### Sphere formation assay

For tumorsphere cultures, the isolated cells at 3000 cells/well were seeded in ultralow attachment 6-well plates (Corning, NY) and cultured for 10 days instem cell medium. RPMI-1640 (Gibco) medium containing 20 ng/ml EGF (Millipore), 20 ng/mL bFGF (Solarbio), B27 (1:50,Gibico) and 4 µg/mL insulin (Solarbio). The cells were exposed to fresh medium every 4 days. The number of spheres (defined as diameter ≥ 70μm) was counted under the microscope, and the sphere-forming efficiency was calculated based on the number of initially seeded cells.

### Total RNA extraction and quantitative real-time PCR

Total RNA of thyroid cancer cells and fresh tissues was extracted by TRIzol reagent (Invitrogen, Carlsbad, CA, USA), and was reversetranscribed with Prime Script RT master Mix (TakaRa, Tokyo, Japan). The reverse transcription of miR-146a was performed with a RevertAid First Strand cDNA Synthesis Kit (Thermo Scientific, USA) according to the instructions. SYBR Premix Ex Taq II (TakaRa, Tokyo, Japan) and specific primers were used to perform quantitativereal-time PCR. The primer sequences are listed in Additional file 2.1.

### Clinical data and tissue samples

The tissue microarrays including the specimen from 136 PTC patients with complete clinicopathological information, 29 ATC patients with prognostic information and 30 non tumor patients who were employed in this study for immunohistochemical staining of KDM1A. All patients were from the Cancer Institute of Tianjin Medical University and the affiliated Hospital of Tianjin Medical University. Twenty-one pairs of matched fresh tissues of PTC carcinoma and adjacent normal thyroid follicular tissue were collected in 2019 for total RNA extraction. This study was performed with the approval of the Tianjin Medical University Institutional and Hospital Ethics Committee.

### Immunohistochemistry (IHC)

The tissue microarrays and tissue sections from tumor xenografts were stained with the indicated antibodies according to the immunohistochemistry protocol. The stained sections were examined as described previously 25.

### Western blotting

Cells were lysed with RIPA lysis buffer (Solarbio), and the protein concentration was quantified by the BCA method. Then the samples were mixed with loading Buffer (Solarbio) for SDS-PAGE. The proteins in the gel were transferred to PVDF membranes (Beyotime) using Bio-Rad's standard wet membrane transfer device, blocked with 5% skimmed milk (Solarbio) and incubated with primary antibody at 4 °C overnight. The next day, after incubation with secondary antibody, the PVDF membrane was subjected to chemiluminescence detection by an ECL Western Blotting Detection Kit (Human IgG) (Solarbio).

### Cell transfection and lentivirus infection

Small interfering RNAs (siRNAs) were directly synthesized (GenePharma, Shanghai, China).The full-length human KDM1A, HIF-1α and KDM1A-K661A sequences were cloned into the pcDNA3.1 vector (Invitrogen, Shanghai, China). siRNAs and pcDNA3.1 were transfected into cells using Lipofectamine 2000 (Invitrogen, Shanghai, China) according to the manufacturer's instructions. 48 h later, the cells were harvested for further experiments.

ShRNAs were delivered by lentiviral infection with lentiviruses produced by transfection of 293T cells with the vector pLKO.1. The sequences of siRNA and shRNA are listed in Additional file 2.2.

### RNA-seq analysis

CAL-62 cells with or without KDM1A were lysed using TRIzol reagent (Invitrogen, Carlsbad, CA, USA). mRNA was isolated following the TRIzol-based RNA extraction method. The global gene expression profiles were detected by mRNA sequencing on the Illumina HiSeq Xten sequencing platform (Majorbio Bio-pharm Technology Co., Ltd, Shanghai, China). Bioinformatics analyses were implemented as described previously 26. The RNA-Seq data has been deposited in the Sequence Read Archive (SRA) under accession number of PRJNA780251.

### Chromatin immunoprecipitation (ChIP) assays

ChIP assay was carried out with a Chromatin Immunoprecipitation Kit (#9005s, Cell Signaling) according to the manufacturer's instructions. The cells were fixed with 1% formaldehyde, and the cross-linked chromatin was sonicated to generate 200-800bp DNA fragments for subsequent immunoprecipitation. Then, the protein/DNA complexes were eluted and analyzed using real-time quantitative PCR. The sequences of ChIP-primer are listed in Additional file 2.3.

### Antibodies and drugs

The antibodies used in this study are listed in Additional file 2.4. Doxorubicin, cycloheximide, MG132, DMOG and GSK-LSD1 were all purchased from Selleck Chemicals (Houston, TX, USA).

### Statistical analysis

All the data presented are from at least three independent biological experiments and are shown as the mean ± SD. An unpaired two-tailed Student's *t* test was used for statistical analysis of the experiments, Kaplan-Meier analysis was employed for survival analysis, and the differences in the survival probabilities were assessed using the log-rank test. Mann-Whitney U test was performed to analyze the difference in protein expression level of normal thyroid tissue, PTC and ATC. *P* < 0.05 was considered to indicate statistical significance. All data were statistically analyzed with GraphPad Prism 6.0 and SPSS 20.0 software.

Detailed information about other experiments is included in Additional file 1.

## Results

### KDM1A is screened out as a histone methylation modifier that which engages in thyroid cancer stemness

**Figure 1:**
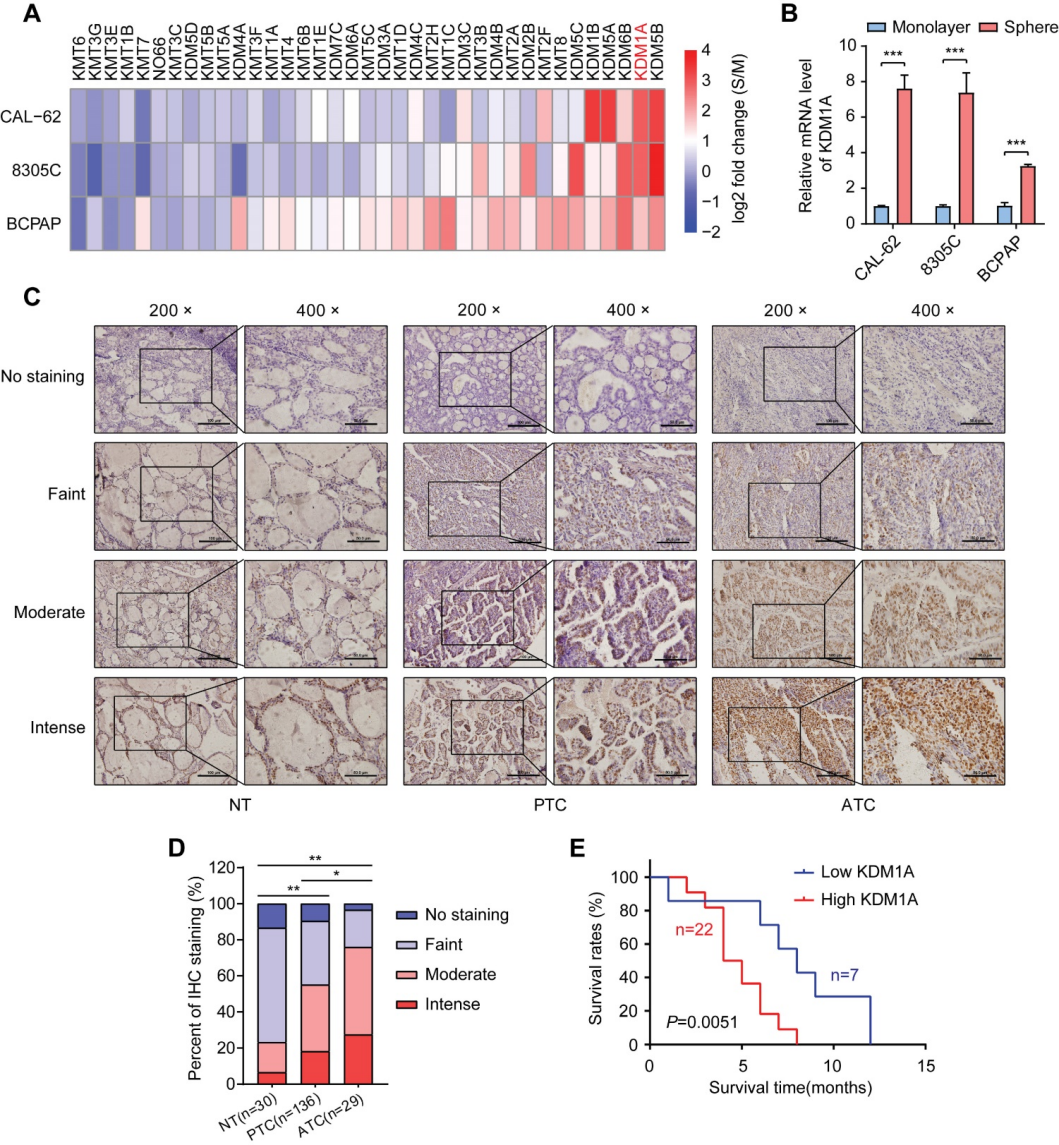
** KDM1A was identified as a stemness-associated histone methylation modifier, and its clinical relevance.** A. Analysis of the mRNA expression levels of 37 members of the histone methylation modifier expressed in CSCs (spheres) or their corresponding non-CSCs (monolayer cells) derived from three thyroid cancer cell lines by quantitative PCR. B. The mRNA expression of KDM1A was significantly higher in CSCs (spheres) than in the corresponding non-CSCs (monolayer cells) derived from three thyroid cancer cell lines. C. Representative immunohistochemical staining of KDM1A in normal thyroid specimens, PTC specimens and ATC specimens. The scale bar in the 200× picture is 100 μm, and the scale bar in the 100 × picture is 50 μm. D. The results of immunohistochemical staining revealed varying protein expression levels of KDM1A in ATC, PTC and normal thyroid specimens. E. Kaplan-Meier analysis of the overall survival (OS) rate in 29 ATC patients with high or low expression of KDM1A, which was measured by immunohistochemical staining. Data are shown as the mean ± SD of three replicates. (*P < 0.05, **P < 0.01, ***P < 0.001)

To identify key histone methylation modifiers in regulating the stemness of thyroid cancer, we utilized a sphere formation assay to enrich CSCs in three thyroid cancer cell lines. As expected, spheroid cells showed much higher mRNA expression levels of stemness-related genes than adherent cells (Figure S1A). Then, we analyzed the mRNA expression levels of 21 histone methyltransferases and 16 histone demethylases, which have been reported to play essential roles in cancer progression (Figure S1B) 27, in spheroids and monolayer thyroid cancer cells by RT-qPCR. Intriguingly, we found that several histone demethylases such as KDM5B, KDM1A, KDM6B, KDM5A and KDM1B, were enriched in thyroid cancer spheres compared to non-CSCs (Figure 1A and Table S1). Previous studies have reported that KDM1A promotes the proliferation, migration and invasion of PTC and serves as an oncogene in PTC 28, 29. However, its impact on the stemness of thyroid cancer is still unclear. Of note, KDM1A was dramatically enriched in three thyroid CSCs (spheres) compared to their corresponding non-CSCs (monolayer cells) (Figure 1B). Therefore, we hypothesize that KDM1A may contribute to the maintenance of thyroid cancer stemness and cancer dedifferentiation.

Next, we examined KDM1A protein expression in tissue samples derived from normal thyroid (30 cases), PTCs (136 cases) and ATCs (29 cases) by IHC staining (Figure 1C). As shown in Figure 1D, we found that the protein expression of KDM1A was higher in cancer tissues than in normal tissues, especially in ATCs. Importantly, high KDM1A expression was significantly correlated with a decreased survival time in ATC (Figure 1E). Then we examined the relationship between KDM1A expression level and some clinical characteristics in 136 patients with PTC, and the results revealed that the protein expression level of KDM1A was positively related to BRAF^V600E^ mutation (*P* = 0.037) and lymph-node metastasis (*P* = 0.015), two poor prognostic characteristics of PTC (Table S2). Multivariate logistic analysis of lymph node metastasis and clinicopathological features showed that KDM1A expression, age and BRAF mutation were independent prognostic factors for lymph node metastasis in PTC (Table S3). Next, we detected the mRNA expression levels of KDM1A in 21 randomly selected PTC tissues and their paired adjacent noncancerous tissues. The results showed that PTC tissues had increased KDM1A mRNA expression (Figure S1C). Similarly, higher mRNA expression of KDM1A in PTC compared to normal thyroid tissues was also found in the TCGA database (Figure S1D). The mRNA expression of KDM1A in PTC tissues and their paired normal tissues also showed this pattern in the TCGA database (Figure S1E). Furthermore, we analyzed its expression in human normal thyroid cells, PTC cells, and ATC cells. Compared to normal thyroid cells, KDM1A was highly expressed in human thyroid cancer cells, especially in ATCs (Figure S1F-G). Taken together, these results suggested that KDM1A played essential roles in the stemness maintenance of thyroid cancer cells, thereby inducing dedifferentiation and promoting thyroid cancer progression.

**Figure 2:**
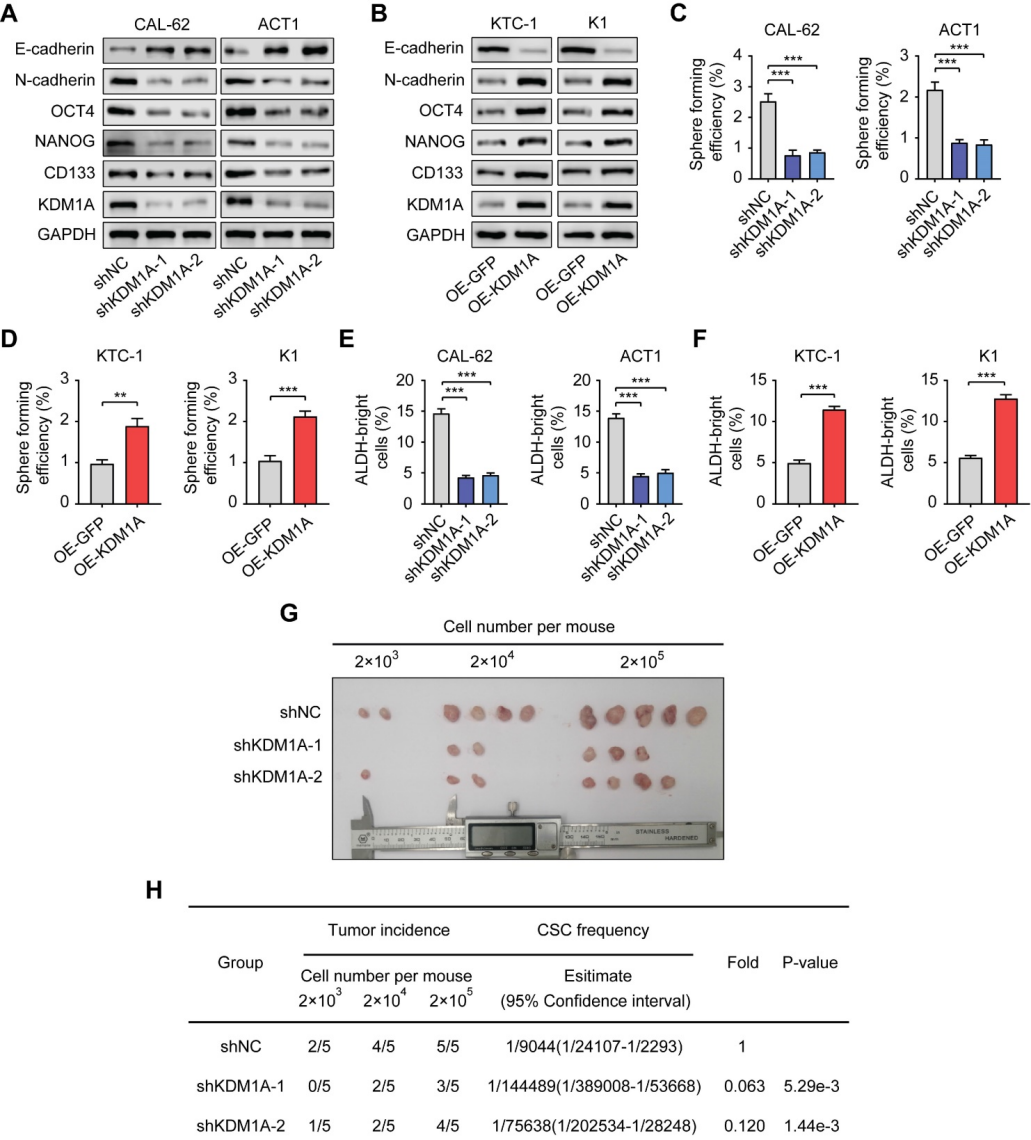
** KDM1A maintains the stemness of thyroid cancer cells.** A, B. The protein expression levels of CSC markers, including NANOG, OCT4 and CD133, and EMT markers, including E-cadherin and N-cadherin, were examined by Western blotting in shKDM1A-transfected ATC cells (A) and KDM1A overexpression plasmid-transfected PTC cells (B). C, D. Representative images of sphere formation induced by the transfection of shKDM1A into CAL-62 and ACT1 cells (C) or the transfection of the KDM1A overexpression plasmid into KTC-1 and K1 cells (D). The surviving colonies were measured for the number of tumorospheres. The scale bar is 100 μm. E, F. ALDEFLUOR assay of ATC cells transfected with shKDM1A (E) and PTC cells transfected with KDM1A overexpression plasmid (F). G, H. CAL-62 cells with or without KDM1A knockdown were subcutaneously injected into the tissues of nude mice at a density of 2 × 10^3^, 2 × 10^4^ or 2 × 10^5^ cells per mouse. Six weeks later, the number of mice that had developed tumors was counted (G). The frequency of CSCs was calculated using ELDA software (H). Data are shown as the mean ± SD of three replicates. (**P < 0.01, ***P < 0.001)

To further confirm the regulation of KDM1A on the stemness properties of thyroid cancer, we used two independent shRNAs to suppress the expression of KDM1A in two ATC cell lines with relatively high endogenous KDM1A expression (CAL-62 and ACT1) (Figure 2A). Moreover, we overexpressed KDM1A in two PTC cell lines with relatively low endogenous KDM1A expression (KTC-1 and K1) (Figure 2B). KDM1A knockdown reduced spheroid formation ability and CSC markers in ATC cells (Figure 2A, Figure 2C and Figure S2C). In contrast, overexpression of KDM1A enhanced the spheroid formation ability and CSC markers of PTC cells (Figure 2B, Figure 2D and Figure S2D). Additionally, we performed ALDEFLUOR assay to detect the activity of ALDH1 as well as CSCs enrichment, monitoring the change in CSC enrichment in thyroid cancer cells after KDM1A knockdown or overexpression. The results showed that the ALDEFLUOR bright population was positively regulated by KDM1A in thyroid cancer cells (Figure 2E and 2F). Furthermore, we also detected the global methylation levels of related histone markers in thyroid cancer cells and found that only the methylation of H3K4me1 and H3K4me2 was regulated by KDM1A (Figure S2A-B). In addition, KDM1A knockdown also inhibited epithelial-mesenchymal transition (EMT), shown by increased E-cadherin and decreased N-cadherin (Figure 2A), and decreased the migration and invasion capabilities of thyroid cancer cells (Figure S3A). Overexpression of KDM1A exhibited the opposite effects (Figure 2B and Figure S3B), suggesting that KDM1A was able toinduce EMT in thyroid cancer cells. To determine whether KDM1A could promotethe stemness of thyroid cancer cells *in vivo*, mice injected with KDM1A knockdown CAL-62 cells. These mice displayed lower tumor volume and tumor-formation ability than control mice, indicating silence of KDM1A dramatically attenuated the stemness of thyroid cancer cells (Figure 2G-H).

### KDM1A induces chemotherapy resistance in thyroid cancer cells

**Figure 3:**
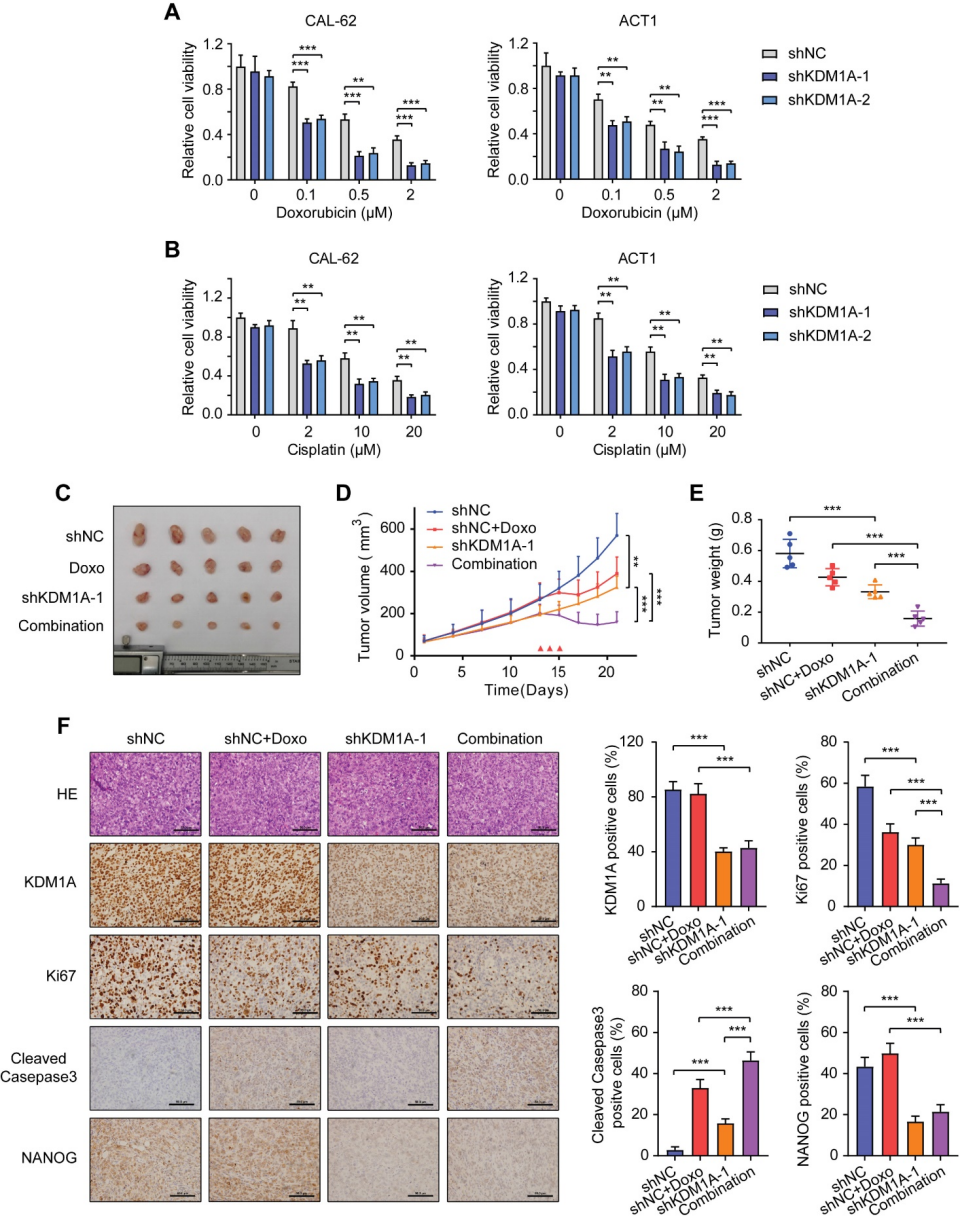
** KDM1A negatively regulates the chemosensitivity of thyroid cancer cells.** A, B. Cell viability of shKDM1A-transfected ATCs after treatment with doxorubicin (A) or cisplatin (B) in different concentration gradients for 48 h. C. Representative images of subcutaneous xenografts in nude mice derived from shKDM1A-transfected CAL-62 cells or shNC-transfected CAL-62 cells that were treated with or without doxorubicin. We started to measure the growth of the tumor every two or three days after the average volume of tumors reached 50 cm^3^. After 12 days of growth, doxorubicin was intraperitoneally injected daily for 3 consecutive days. *n* = 5 mice per group. The subcutaneous xenografts were dissected and are shown on day 21. D. Growth curves of the subcutaneous xenografts in each group. E. Analysis of the tumor weight of the xenografts in each group. F. The expression levels of KDM1A, Ki67, cleaved caspase 3 and NANOG in xenografts of each group were assessed by immunohistochemical staining. Unpaired two-tailed Student's *t*-test was used to analyzed the difference of positive staining cells ratio between different groups. The scale bar is 50 μm. Data are shown as the mean ± SD of three replicates. (**P < 0.01, ***P < 0.001)

Since CSC is a principal factor that determines the response to chemotherapy, we asked whether KDM1A could affect the sensitivity of thyroid cancer to chemotherapy. We selected doxorubicin, the only cytotoxic agent that is approved by the United States FDA as single-drug chemotherapy for ATC treatment 30 to conduct subsequent experiments. The results showed that KDM1A knockdown reinforced the inhibitory effect of doxorubicin on ATC at various concentrations (Figure 3A). KDM1A overexpression enhanced the resistance of PTC todoxorubicin treatment (Figure S4A). KDM1A knockdown also increased the ability of doxorubicin to induce the apoptosis of ATC cells (Figure S4B). In addition to doxorubicin, ATC cells with KDM1A knockdown showed increased responses to cisplatin (Figure 3B) and sorafenib (Figure S4C). Therefore, silencing KDM1A may sensitize thyroid cancer to chemotherapy.

To further verify the effect of KDM1A on chemosensitivity *in vivo*, we established a xenograft model by subcutaneously injecting CAL-62 ATC cells into nude mice before doxorubicin treatment. Although either KDM1A knockdown or doxorubicin treatment alone resulted in partial tumor regression, combined KDM1A knockdown with doxorubicin treatment was able to further inhibit tumor growth (Figure 3C-E). The immunohistochemical staining of xenografts confirmed that shKDM1A suppressed the protein expression of KDM1A and NANOG (Figure 3F). In addition, the combination of shKDM1A and doxorubicin led to a lower degree of tumor proliferation and more apoptosis, as determined by Ki67 and cleaved caspase 3 staining, respectively (Figure 3F). In conclusion, KDM1A attenuates the chemosensitivity of thyroid cancer, and combining KDM1A inhibition with chemotherapy can achieve stronger antitumor effects.

### KDM1A silencing attenuates the activity of the Wnt/β-catenin signaling pathway

**Figure 4:**
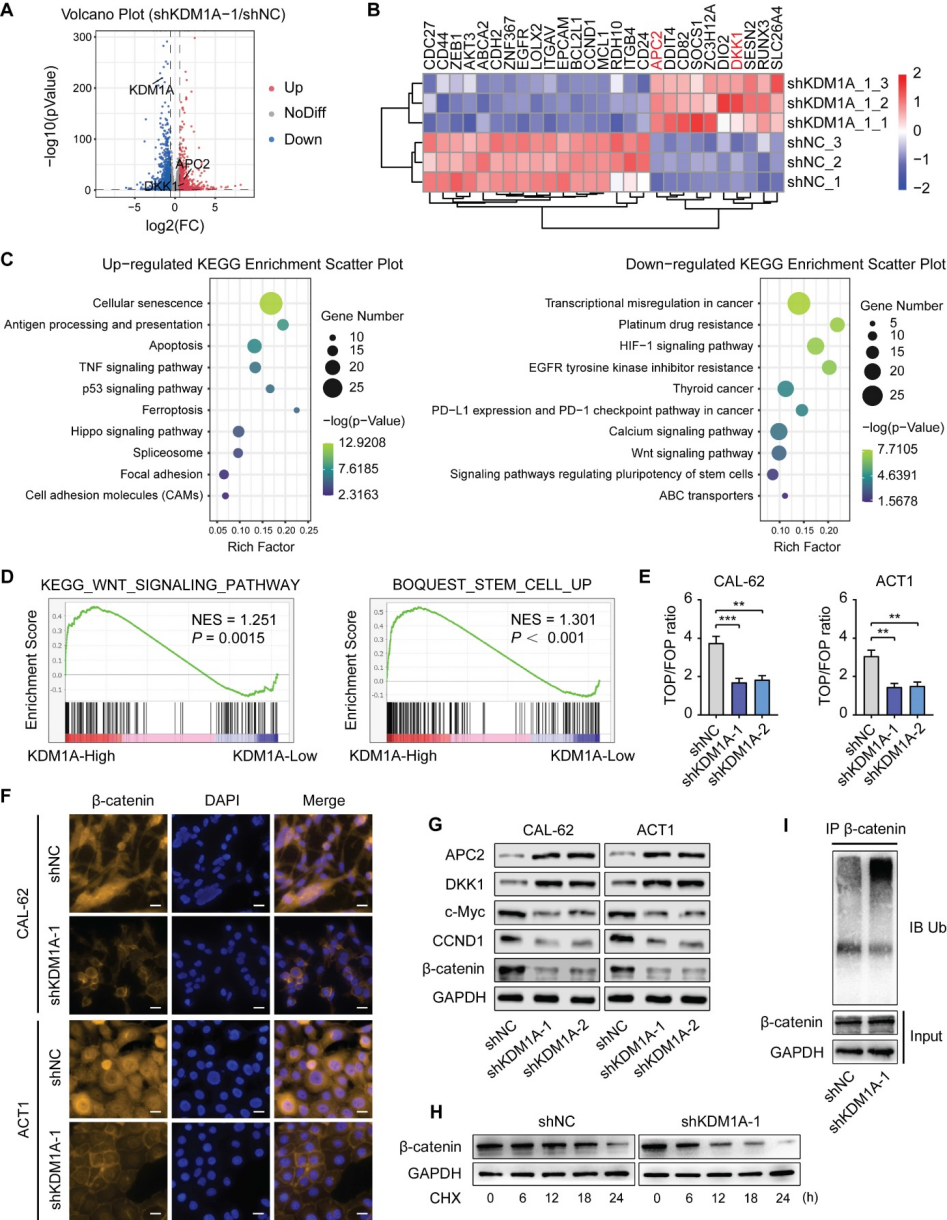
** KDM1A knockdown attenuates the activity of the Wnt/β-catenin signaling pathway.** A. Volcano plot of the RNA-seq analysis of CAL-62 cells after transfection with shKDM1A. B. Representative gene expression in CAL-62 cells after transfection with shKDM1A.These differentiallyexpressedgenes are widely involved in apoptosis (MCL1, BCL2L1), EMT (ZEB1, CDH2, ZNF367, LOXL2, ITGAV, RDH10, CD82, RUNX3), cell proliferation (CDC27, AKT3, CCND1, EGFR, ITGB4, DDIT4, SOCS1, APC2, DKK1, SESN2),drug resistance (ABCA2), thyroid differentiation (DIO2, SLC26A4), immune escape (ZC3H12A) and maintenance of stem cells (CD44, EPCAM, CD24). C. Representative KEGG pathway analysis of up-regulated and down-regulated genes after transfection with shKDM1A. D. GSEA showed that genes differentially expressed after KDM1A knockdown were significantly enriched in the gene sets related to the Wnt signaling pathway and stemness. E. A TOP/FOP luciferase reporter assay was employed to detect the transcriptional activity of the canonical Wnt signaling pathway in ATCs after transfection with shKDM1A. F. Immunofluorescence of ATCs with or without KDM1A knockdown for β-catenin (orange) and 4′,6-diamidino-2-phenylindole (DAPI) (blue). The scale bar is 20 μm. G. Western blotting validation of differential gene expression related to the Wnt/β-catenin signaling pathway in ATC cells with KDM1A knockdown. H. The protein expression of β-catenin in CAL-62 cells treated with 100 μM CHX for different durations was measured by western blot analysis. I. KDM1A knockdown increased the endogenous ubiquitination of β-catenin.CAL-62 cells with or without KDM1A knockdown were treated with MG132 (20 μM) for 6 h. Then, the total and ubiquitinated β-catenin levels were analyzed by Western blotting. Data are shown as the mean ± SD of three replicates. (**P < 0.01, ***P < 0.001)

To further elucidate the underlying mechanism of KDM1A's carcinogenic effect in thyroid cancer, RNA-sequencing was applied to screen the potential downstream genes regulated by KDM1A. KDM1A knockdown in CAL-62 cells resulted in more than 1.5-fold up-regulation of 855 genes and more than 1.5-fold down-regulation of 1076 genes (Figure 4A). These differentially expressed genes are widely involved in various biological behaviors of cancers, including apoptosis, EMT, cell proliferation, maintenance of stem cells and so on (Figure 4B). We performed Kyoto Encyclopedia of Genes and Genomes (KEGG) pathway analysis (Figure 4C) and Gene Ontology (GO) enrichment analysis (Figure S5A)of the differentially expressed genes. The results showed that multiple crucial signaling pathways and biological processes involved in tumorigenesis were regulated by KDM1A, suggesting that KDM1A plays essential roles in thyroid cancer development.

Given the important roles of the Wnt pathway in regulating the stemness of cancer 31 and since both GO and KEGG analysis of our RNA-seq results revealed the down-regulation of the Wnt/β-catenin signaling pathway after KDM1A knockdown, we hypothesized that KDM1A regulated the stemness and chemosensitivity of thyroid cancer through this pathway. We carried out GSEA to confirm that KDM1A is positively related to the Wnt signaling pathway and stem cell maintenance (Figure 4D). A TCF/LEF reporter assay, which specifically measures the transcriptional activity of the Wnt pathway, was also employed to confirm that KDM1A can regulate the Wnt pathway (Figure 4E and Figure S6A). Moreover, we performed immunofluorescence staining of β-catenin in ATC and PTC cells. The results showed that nuclear β-catenin was reduced in ATC cells after KDM1A knockdown (Figure 4F) and was augmented in PTC cells after KDM1A overexpression (Figure S6D). Meanwhile, the expression levels of CCND1 and c-Myc, two acknowledged downstream genes of the Wnt pathway were also regulated by KDM1A (Figure 4G and Figure S5B, S6B-C). Although the total protein expression level of β-catenin was dramatically regulated by KDM1A (Figure 4G, Figure S6B), the expression level of CTNNB1, the mRNA encoding the protein of β-catenin, was not markedly modulated by KDM1A (Figure S5B and S6C).

To understand the molecular mechanisms by which KDM1A regulates the Wnt/β-catenin pathway, we analyzed the RNA-seq data for genes associated with this pathway and found that the expression levels of APC2 and DKK1, which serve as antagonists of the Wnt/β-catenin pathway were significantly increased after KDM1A knockdown (Figure 4B). Then we confirmed that the protein and mRNA expression of APC2 and DKK1 were significantly increased upon KDM1A knockdown (Figure 4G and Figure S5B) and were decreased after KDM1A overexpression (Figure S6B-C). Furthermore, our *in vivo* model also suggested that the expression levels of both APC2 and DKK1 obviously increased after KDM1A inhibition (Figure S5C).

It is well known that both APC2 and DKK1 can promote the degradation of β-catenin by enhancing its ubiquitination 32, 33; therefore, we detected the stability of β-catenin and the endogenous ubiquitination level of β-catenin in CAL-62 cells with or without KDM1A knockdown. CAL-62 cells were treated with cycloheximide (CHX), a protein synthesis inhibitor, and subjected to western blotting analysis (Figure 4H) showed lower stability of β-catenin in CAL-62 cells with KDM1A knockdown. In addition, treatment of CAL-62 cells with MG132, a proteasome inhibitor, restored KDM1A knockdown-induced β-catenin reduction and obviously increased the level of protein ubiquitination (Figure 4I). In summary, KDM1A can upregulate the Wnt/β-catenin signaling pathway in thyroid cancer cells by suppressing the ubiquitin-proteasome degradation of β-catenin and its regulation on APC2 and DKK1 may contribute to this process.

### KDM1A regulates the Wnt/β-catenin signaling pathway through APC2 and DKK1

**Figure 5:**
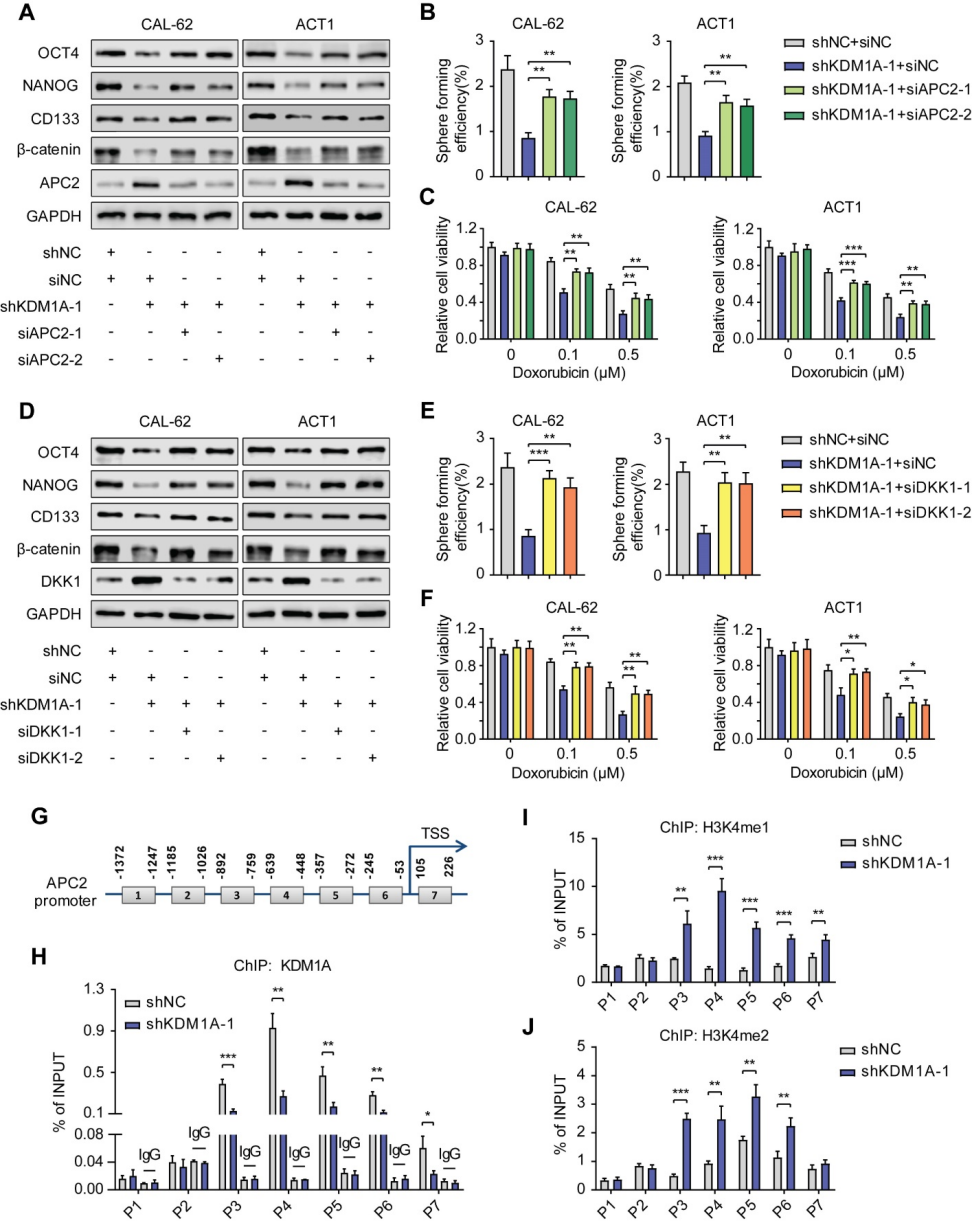
** KDM1A regulates the Wnt/β-catenin signaling pathway through APC2 and DKK1.** A, D. Western blotting validated that suppressing the expression of APC2 (A) or DKK1 (D) by siRNA rescued the reduced protein expression levels of CSC markers and β-catenin induced by KDM1A knockdown in ATC cells. B, E. Repressing APC2 (B) or DKK1 (E) with siRNA restored the attenuated sphere formation ability caused by KDM1A knockdown in ATC cells. C, F. Repressing APC2 (C) or DKK1 (F) by siRNA reverted the increased response to doxorubicin in ATC cells with KDM1A knockdown. G. Schematic of the ChIP primers designed for the APC2 promoter regions (1-7). H, I, J. The enrichment levels of KDM1A (H), H3K4me1 (I) and H3K4me2 (J) in different regions of the APC2 gene promoter in CAL-62 ATC cells with or without KDM1A knockdown were determined by ChIP assay. Data are shown as the mean ± SD of three replicates. (*P < 0.05,**P < 0.01, ***P < 0.001)

To further confirm that the Wnt pathway is enhanced by KDM1A via its regulation on APC2 and DKK1, we transfected siRNAs against APC2 or DKK1 into the KDM1A-silenced ATC cells. The results showed that suppression of either APC2 or DKK1 could rescue the altered phenotypes in ATC cells caused by KDM1A knockdown, such as the down-regulated protein expression levels of β-catenin and CSC markers (Figure 5A and 5D), the attenuated spheroid formation ability (Figure 5B and 5E) and the increased response to chemotherapy (Figure 5C and 5F).

Given that previous studies have shown that KDM1A affects gene expression by regulating the histone methylation level of the promoter, we investigated whether KDM1A can directly bind to the promoters of APC2 and DKK1 to inhibit their transcription. Primers sets spanning the proximal promoter regions of APC2 (Figure 5G) and DKK1 (Figure S7A), from approximately -1,400 to +300 bp relative to the transcriptional start sites (TSSs), were designed for ChIP-qPCR. The results showed that KDM1A could bind to the APC2 promoter but not the DKK1 promoter (Figure 5H, Figure S7B), indicating that DKK1 was an indirect target of KDM1A. In addition, we also analyzed the enrichment of H3K4me1 and H3k4me2 in the promoter of APC2 and DKK1. Consistent with the KDM1A ChIP-qPCR results,the enrichment of H3K4me1/2 in the APC2 promoter but not the DKK1 promoter was markedly increased after KDM1A knockdown (Figure 5I-J, Figure S7C-D). These results suggest that APC2 is a direct target of KDM1A and is regulated by KDM1A via demethylation of H3K4me1 and H3K4me2, while DKK1 is an indirect target of KDM1A.

### KDM1A decreasesDKK1 expression by stabilizing the HIF-1α protein

**Figure 6:**
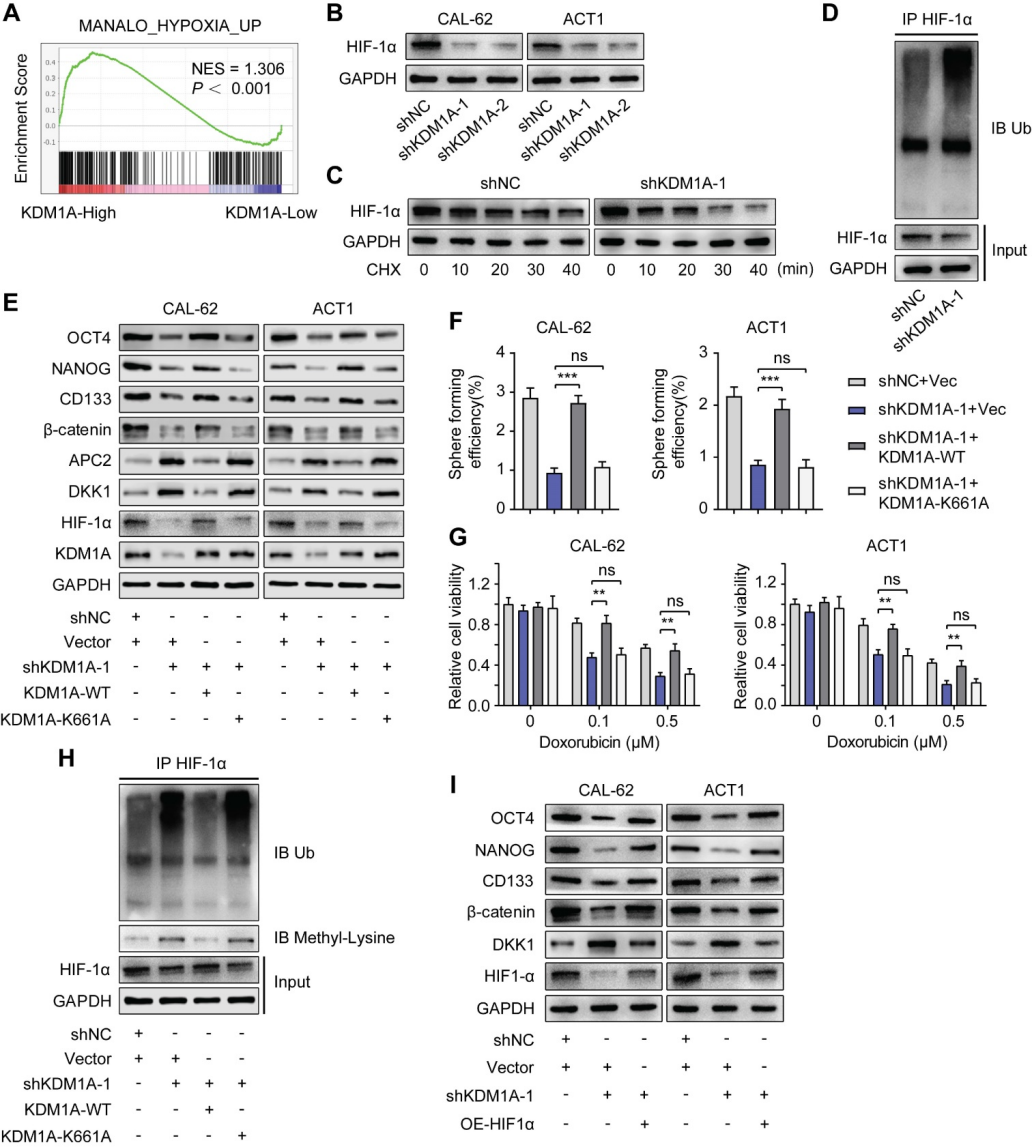
** KDM1A stabilization of HIF-1α is responsible for its regulation of the Wnt pathway.** A. GSEA showed that genes differentially expressed after KDM1A knockdown were significantly enriched in gene set-related hypoxia. B. The protein expression of HIF-1α was examined by Western blotting in KDM1A knockdown ATC cells. C. The protein expression of HIF-1α in CAL-62 cells treated with 100 μM CHX for different durations was measured by western blot analysis. The cells were pretreated with DMOG (500 μM), a cell-permeable and competitive inhibitor of HIF-PH that results in HIF-1α stabilization and accumulation, for 24 h before incubation with CHX. D. KDM1A knockdown increased the endogenous ubiquitination of HIF-1α. CAL-62 cells with or without KDM1A knockdown were treated with MG132 (20 μM) for 6 h. Then, total and ubiquitinated HIF-1α levels were analyzed by Western blotting. E. Overexpression of KDM1A-WT, but not KDM1A-K661A, restored the decreased protein expression levels of HIF-1α, CSC markers and β-catenin and increased the protein expression levels of APC2 and DKK1 in KDM1A-depleted ATC cells. F. Quantification of the sphere formation of KDM1A-depleted ATCs after introducing KDM1A-WT or KDM1A-K661A. G. Overexpressing KDM1A-WT instead of KDM1A-K661A reversed the increased response to doxorubicin in KDM1A knockdown ATC cells. H. Introduction of KDM1A-WT instead of KDM1A-K661A to KDM1A knockdown CAL-62 cells reversed the up-regulation of endogenous ubiquitination and HIF-1α protein methylation. CAL-62 cells with or without KDM1A knockdown were treated with MG132 (20 μM) for 6 h. Then, total and ubiquitinated HIF-1α levels were analyzed by Western blotting. I. Western blotting validated that the introduction of HIF-1α to ATC cells reverses the decreased protein expression of β-catenin and CSC markers and the increased protein expression of DKK1 induced by KDM1A knockdown. Data are shown as the mean ± SD of three replicates. (**P < 0.01, ***P < 0.001)

A previous study reported that KDM1A could suppress the expression of DKK1 in colorectal carcinoma 34. However, the mechanism of this suppression remains unknown. Notably, KEGG and GO analysis of our RNA-seq data revealed that KDM1A knockdown restrained the HIF1 signaling pathway and the response to hypoxia. GSEA also validated that KDM1A facilitated hypoxia (Figure 6A). It has been reported that the activity of the Wnt pathway and its regulation on pluripotency are affected by hypoxia 35. Therefore, we hypothesized that KDM1A inhibited DKK1 expression through the HIF-1α pathway. The protein expression of HIF-1αwas significantly affected by KDM1A in thyroid cancer cells (Figure 6B and Figure S8A), but the mRNA level of HIF-1α was not regulated by KDM1A (Figure S8B-C). A previous study reported that KDM1A suppressed HIF-1α protein expression by facilitating its ubiquitin-proteasome degradation 36, so we detected the stability of HIF-1α and the endogenous ubiquitination level of HIF-1α in CAL-62 cells with or without KDM1A knockdown. Intriguingly, we uncovered that the stability of HIF-1α in CAL-62 cells with KDM1A knockdown was weaker (Figure 6C). In addition, the reduction in HIF-1α caused by shKDM1A could be rescued by treatment with MG132 and KDM1A knockdown led to an obvious augmentation of the endogenous ubiquitination of HIF-1α (Figure 6D).

**Figure 7:**
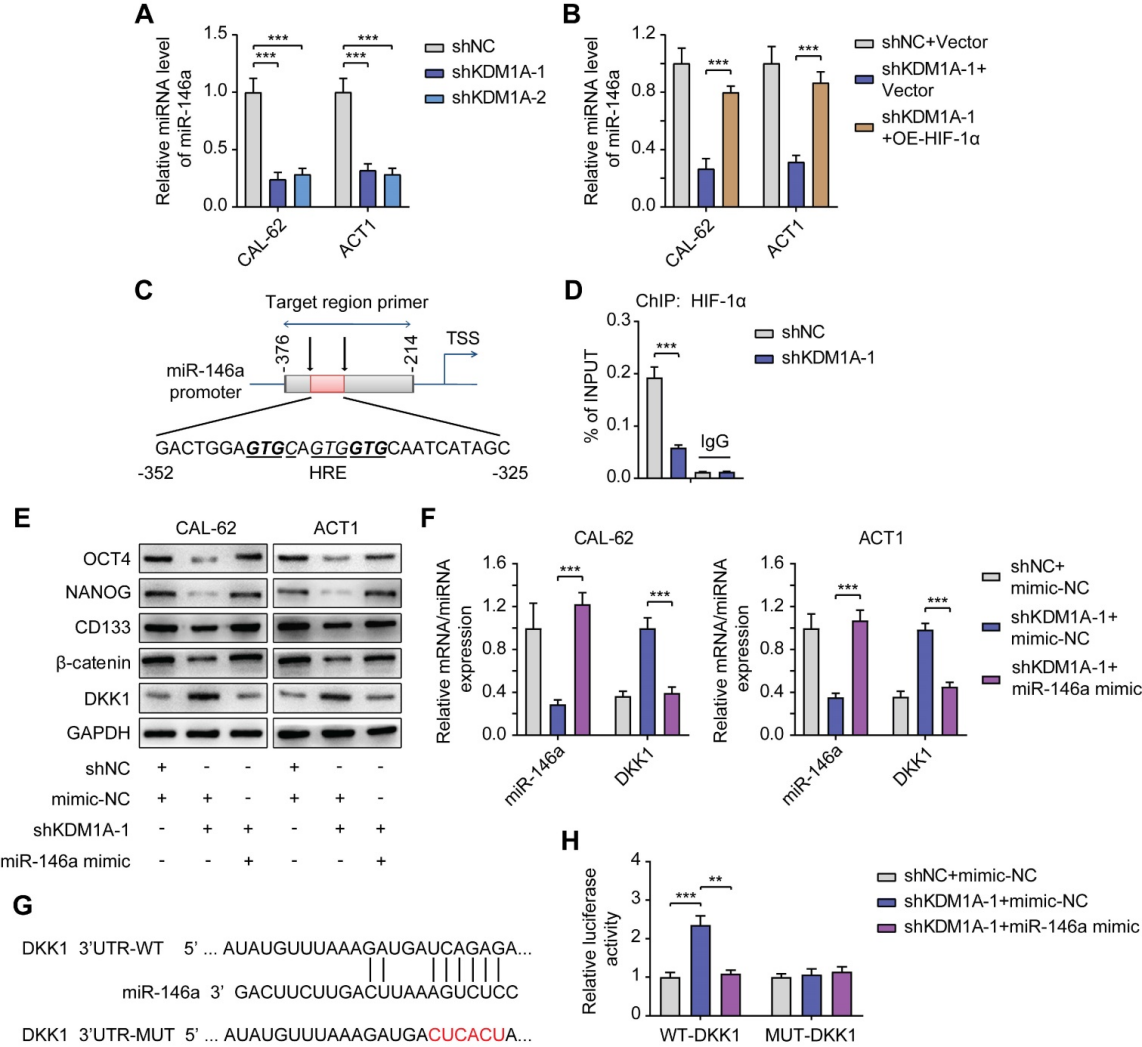
** KDM1A regulates DKK1 expression via the HIF-1α/microRNA-146a axis.** A. The relative expression levels of miR-146a were measured by qPCR in shKDM1A-transfected ATC cells. B. Quantitative PCR proved that overexpression of HIF-1α in ATC cells can revert the decreased miR-146a expression caused by KDM1A knockdown. C. Schematic of the ChIP primers designed for miR-146a promoter regions to analyze the HIF-1α binding affinity. The putative HIF-1α binding site (HRE) identified on the miR-146a promoter is also displayed. D. The enrichment of HIF-1α in the promoter region of miR-146a was detected by ChIP-qPCR. E. Western blotting validated that the introduction of the miR-146a mimic to ATC cells could restore the decreased β-catenin and CSC marker expression induced by KDM1A knockdown. F. Quantitative PCR validated that the introduction of the miR-146a mimic to ATC cells could restore the increased DKK1 expression induced by KDM1A knockdown. G. Bioinformatic alignment analysis of miR-146a and the 3'UTR of DKK1. The putative binding sequences and mutant sites are also displayed. H. The binding relationship between miR-146a and the DKK1 promoter was analyzed by dual-luciferase reporter assay. Data are shown as the mean ± SD of three replicates. (**P < 0.01, ***P < 0.001)

KDM1A was first identified as a histone demethylase. However, recent studies have shown that KDM1A can also demethylate nonhistone proteins such as HIF-1α 36. KDM1A can demethylate HIF-1α to prevent it from being degraded via the ubiquitin-proteasome pathway. To verify this mechanism in thyroid cancer, we constructed a KDM1A plasmid containing a K661A point mutation (i.e., a lysine-to-alanine mutation at lysine 661) to disrupt its demethylase activity. Then, we transfected KDM1A-depleted ATC cells with a KDM1A-wild-type plasmid or a KDM1A-K661A plasmid to ectopically express KDM1A-WT or KDM1A-K661A, respectively. As the results showed, reintroducing KDM1A-WT but not KDM1A-K661A rescued the decreased protein expression of HIF-1α, β-catenin and CSC markers, increased the protein expression of APC2 and DKK1 (Figure 6E), attenuated spheroid formation ability (Figure 6F) and increased the sensitivity to doxorubicin (Figure 6G) in KDM1A-depleted ATC cells. The augmented ubiquitylation and protein methylation level of HIF-1α caused by KDM1A knockdown in CAL-62 cells could also be rescued by reintroducing KDM1A-WT instead of KDM1A-K661A (Figure 7H). These results implied that KDM1A can demethylate HIF-1α and prevent it from degrading via the ubiquitin-proteasome pathway in thyroid cancer and that its demethylase function is indispensable for thyroid tumorigenesis.

Next, we transfected KDM1A knockdown ATC cells with a HIF-1α overexpression plasmid to validate the effect of HIF-1α, which is regulated by KDM1A, on the Wnt pathway and stemness. As the results showed, overexpressing HIF-1α could reverse the effects generated by KDM1A knockdown in ATC cells, such as decreasing CSC markers and β-catenin (Figure 6I), attenuating spheroid formation ability (Figure S8D) and increasing the response to doxorubicin (Figure S8E). More importantly, we found that the increased DKK1 expression in ATC cells with KDM1A knockdown can also be reverted by introducing HIF-1α (Figure 6I and S8F). Taken together, these data indicated that KDM1A regulated DKK1 expression and that the Wnt pathway was at least partly dependent on HIF-1α.

### KDM1A regulates DKK1 expression via the HIF-1α/microRNA-146a axis

Previous studies have shown that miR-146a is a new target of HIF-1α and that miR-146a can inhibit DKK1 mRNA expression by binding to its 3'UTR 37. We further explored whether KDM1A regulated DKK1 expression through the HIF-1α/microRNA-146a axis in thyroid cancer.

First, we detected decreased miR-146a expression in ATC cells with KDM1A knockdown (Figure 7A) and increased miR-146a expression in PTC cells with KDM1A overexpression (Figure S9A). The decrease in miR-146a expression in ATC cells with KDM1A knockdown can also be rescued by introducing HIF-1α, indicating that miR-146a is a downstream of HIF-1α (Figure 7B). Then, ChIP-qPCR was performed to validate the enrichment of HIF-1α in the promoter of miR-146a and the enrichment was down-regulated upon KDM1A knockdown (Figure 7D). Subsequently, we transfected KDM1A knockdown ATC cells with miR-146a mimic to validate the effect of miR-146a on DKK1. As the results showed, introducing the miR-146a mimic to KDM1A knockdown ATC cells reversed its increased DKK1 expression (Figure 7E-F), decreased the expression levels of the CSC markers and β-catenin (Figure 6E), attenuated the spheroid formation ability (Figure S9B) and increased the response to doxorubicin (Figure S9C). Conversely, transfection of KDM1A-overexpressing PTC cells with a miR-146a inhibitor rescued the decreased DKK1 expression (Figure S9D-E), increased the expression levels of the CSC markers and β-catenin (Figure S9D), and enhanced the spheroid formation ability (Figure S9F) and the resistance to doxorubicin (Figure S9G). To confirm that miR-146a induces the degradation of DKK1 mRNA, a dual-luciferase reporter assay was conducted by cotransfecting WT-DKK1-3'UTR or MUT-DKK1-3'UTR reporter and miR-146a into CAL-62 cells with KDM1A knockdown. Transfection of the miR-146a mimic markedly reversed the enhanced luciferase activity caused by KDM1A knockdown in the WT-DKK1-3'UTR reporter system but not in the MUT-DKK1-3'UTR reporter system (Figure 7H), suggesting that DKK1 was a direct target of miR-146a.

All these results proved that KDM1A can regulate the transcription of DKK1 via HIF-1α/miR-146a axis; and then activate the Wnt pathway to promote the stemness and chemotherapeutic resistance of thyroid cancer.

### Pharmacological inhibition of KDM1A shows antitumor efficiency in thyroid cancer

**Figure 8:**
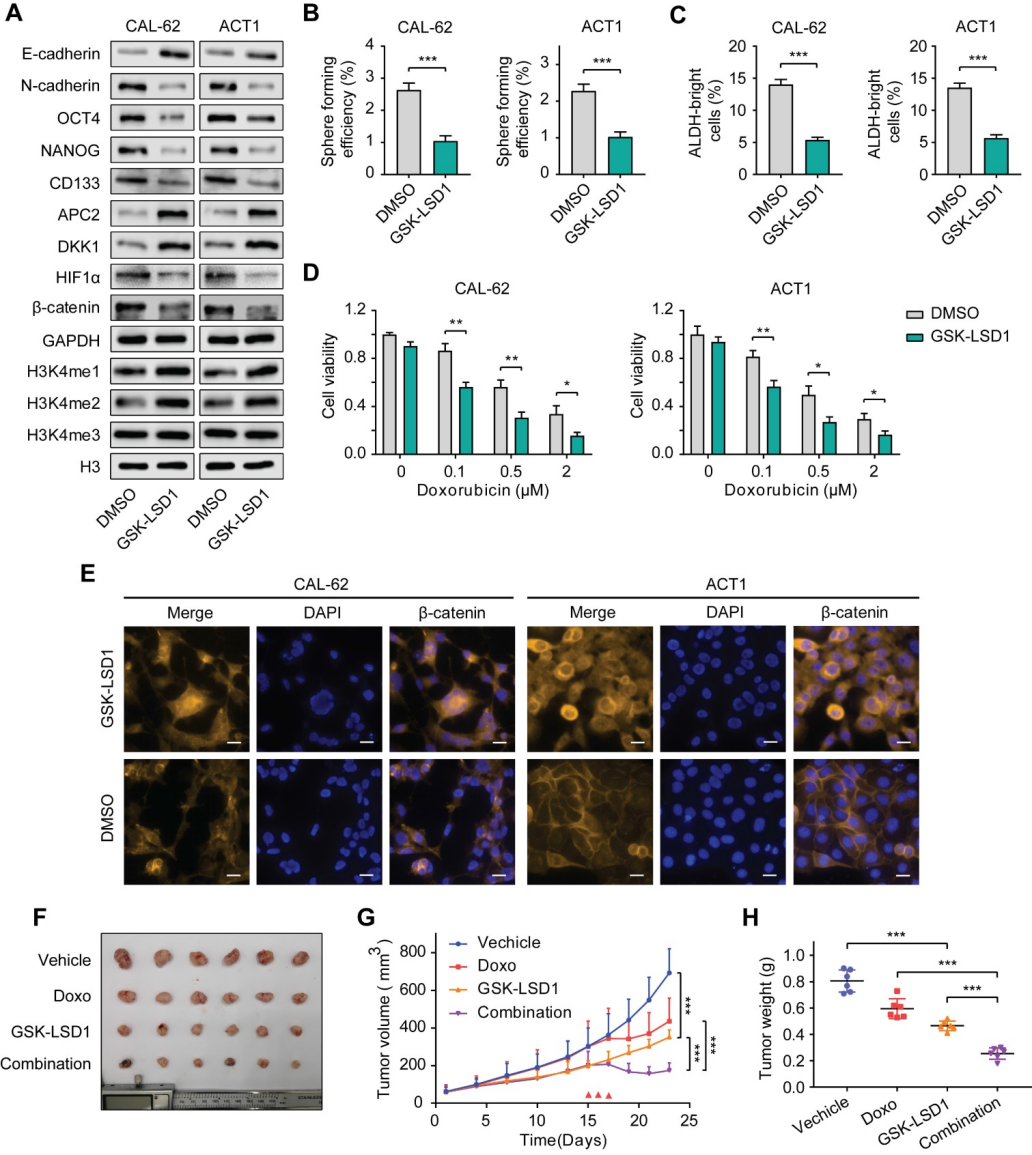
** Pharmacological inhibition of KDM1A shows antitumor efficiency in thyroid cancer.** A. The protein expression levels of CSC markers, EMT markers and H3K4me1/2/3 in ATCs that had been treated with GSK-LSD1 (1 μM) for 5 days were analyzed by western blotting. B. Representative images of sphere formation of ATCs that had been treated with GSK-LSD1 (1 μM) for 5 days. C. ALDEFLUOR assay of ATCs that had been treated with GSK-LSD1 (1 μM) for 5 days. D. The combination of GSK-LSD1 and doxorubicin effectively reduced the viability of ATCs *in vitro*. ATCs were pretreated with GSK-LSD1 (1 μM) for 5 days before the addition of different concentrations of doxorubicin for two more days. E. β-catenin in ATCs that had been exposed to GSK-LSD1 (1 μM) for 5 days was detected by immunofluorescence. The scale bar is 20 μm. F. Representative images of subcutaneous xenografts in nude mice derived from CAL-62 cells that were treated with either GSK-LSD1, doxorubicin or the combination of GSK-LSD1 and doxorubicin. *n* = 6 mice per group. The subcutaneous xenografts were dissected and are shown on day 23. Dosing was performed by intraperitoneal injection once a day at 1 mg/kg for GSK-LSD1 from day 2 to day 20 and 2 mg/kg for doxorubicin from day 15 to day 17. G. Growth curves of the subcutaneous xenografts in each group. H. Analysis of the tumor weight of the xenografts in each group. Data are shown as the mean ± SD of three replicates. (*P < 0.05,**P < 0.01, ***P < 0.001)

Many studies have been devoted to seeking highly selective and active inhibitors targeting KDM1A as antineoplastic drugs due to its multiple carcinogenic effects in various cancers 38-40. We selected GSK-LSD1, a highly selective inhibitor targeting KDM1A to investigate its antitumor effects in thyroid carcinoma. The results showed the protein expression of CSCs markers in CAL-62 and ACT1 cells were dramatically down-regulated by GSK-LSD1 (Figure 8A). Correspondingly, GSK-LSD1 also significantly inhibited spheroid formation in thyroid cancer cells (Figure 8B-C). Consistent with KDM1A knockdown, pretreatment with GSK-LSD1also significantly increased the response of ATC cells to chemotherapy (Figure 8D and Figure S10A). Moreover, treatment with GSK-LSD1 resulted in up-regulation of the global methylation levels of H3K4me1/2 (Figure 8A) and the protein expression levels of APC2 and DKK1 (Figure 8A) as well as the suppression of HIF-1α and β-catenin (Figure 8A and8E). In addition, the migration and invasion abilities of ATC cells were attenuated after treatment with GSK-LSD1 (Figure S10B), which corresponded to the increase in E-cadherin and the decrease in N-cadherin protein expression (Figure 8A).

Next, we sought to investigate the antitumor activity of GSK-LSD1 in thyroid cancer *in vivo*. After ATC xenograft models were established, mice were treated with either GSK-LSD1 or doxorubicin alone, or with both GSK-LSD1 and doxorubicin in combination. The results showed that GSK-LSD1 could significantly delay tumor growth, while the combination with chemotherapeutic agents had a stronger effect in inhibiting thyroid cancer progression (Figure 8F-H). We also detected the weight of mice during the treatment and found that GSK-LSD1 combination in with doxorubicin had little effect on bodyweight compared to doxorubicin alone (Figure S11A). Immunohistochemical staining of xenografts showed that the protein expression levels of Ki67, NANOG and β-catenin were reduced and that the protein expression levels of cleaved caspase-3, APC2 and DKK1 were increased in tumors treated with GSK-LSD1 (Figure S11B). Moreover, co-treatment with GSK-LSD1 and doxorubicin led to greater changes in Ki67 and cleaved caspase-3 protein expression than monotherapy (Figure S11B). All these data suggest that pharmacological inhibition of KDM1A by GSK-LSD1 can inhibit thyroid cancer progression and sensitize it to chemotherapy, and therefore, targeting KDM1A may bea novel therapeutic strategy for patients with advanced thyroid cancer.

## Discussion

In this study, we identified KDM1A as a stemness-associated histone methylation modifier in thyroid cancer that is related to the stem cell pluripotency and chemosensitivity of thyroid cancer *in vitro* and *in vivo*. The underlying mechanism is that KDM1A can demethylate the mono- and dimethylated H3K4 of the APC2 promoter region as well as the nonhistone substrate HIF-1α, which causes the inhibition of APC2 transcription and the activation of the HIF-1α/microRNA-146a/DKK1 axis, which subsequently activates canonical Wnt signaling pathway. Although previous studies have reported that KDM1A activates the Wnt pathway by inhibiting the transcription of its antagonists, APC, Prickle1 and SFRP5 41, we found new mechanisms by which KDM1A regulates the Wnt pathway. Our study greatly enriches the understanding of the mechanisms by which KDM1A plays a role in regulating the Wnt pathway. More importantly, we proposed a new treatment method targeting CSCs for advanced thyroid cancer.

The chromatin immunoprecipitation-sequence data sets of KDM1A and H3K4me1/2 from K562 cells in the ENCODE Epigenomic Data Base revealed that the promoter of APC2 was enriched with KDM1A, but not H3K4me1/2, suggesting that APC2 was a potential target of KDM1A. Our study first confirmed that KDM1A can demethylate H3K4me1/2 of the APC2 promoter region, thereby inhibiting its transcription and activating the Wnt pathway. More importantly, our study first revealed the mechanisms of KDM1A's regulation of DKK1, which has not been elucidated before. In addition, the previous work about KDM1A in thyroid cancer was only focused on PTC, our study for the first time extended the research topic to ATC and uncovered its expression pattern among different thyroid cancer subtypes, this could provide potential biomarkers for identification of highly aggressive thyroid cancer. Importantly, our study also identified key histone methylation modifiers regulating thyroid cancer stemness and highlighted that targeting histone methylation modification in combination with chemotherapy could obviously abolish thyroid cancer progression in mouse model and serve as a new therapeutic strategy for advanced thyroid cancer.

In a variety of tumors with different tissue origins, KDM1A has been reported to regulate stemness and drug resistance, such as hepatocellular carcinoma 10, 41, leukemia 42, and breast cancer 43, though the exact mechanisms are diverse. We were the first to discover and verify that KDM1A is involved in the regulation of stemness and chemosensitivity in thyroid carcinoma and demonstrate that KDM1A activates the Wnt pathway by inhibiting APC2 and DKK1 transcription through its demethylation function on histones of the APC2 promoter region and nonhistone HIF-1α. It's noticeable that KDM1A can also demethylate nonhistone substrates and its regulation of HIF-1α is achieved by directly demethylating the HIF-1αprotein. Many studies have also reported that KDM1A can regulate gene expression independently of its histone demethylation function. It can also demethylate nonhistone proteins 44, 45, while it was first identified as a histone demethylase. Therefore, it is worth paying more attention to the nonhistone substrates of such histone methylation modifiers.

The most dominat pathogenic mechanism in thyroid cancer is the activation of the MAPK and PI3K/Akt pathways caused by BRAF^V600E^ mutation, RAS mutation or RET mutation 46. However, the canonical Wnt signaling pathway also plays an irreplaceable role 47. In high-functioning adenomas and FTC, β-catenin is localized to the plasma membrane just as it is in the normal thyroid gland. In PTC, there is an accumulation of β-catenin in the cytoplasm, while in poorly differentiated and undifferentiated thyroid carcinoma, β-catenin is transferred to the nucleus due to mutations in the β-catenin gene or other genes in the Wnt pathway 48. Indeed, the accumulation of tumor mutation load is also responsible for the development of thyroid carcinoma from low-grade PTC to highly malignant ATC 46. Previous studies also proved that RET/PTC rearrangement could stabilize β-catenin by phosphorylation of a residue outside the GSK3β Ser/Thr domain 49 and that Akt-MAPK-dependent inhibition of GSK3β can also stabilize β-catenin 47. The occurrence of these events leads to an increase in β-catenin in the nucleus, where it can activate oncogenic transcription. Therefore, targeting the Wnt pathway for thyroid cancer therapy may also be a promising strategy.

Apart from the Wnt pathway and stem cell pluripotency, KDM1A can also influence many other important signaling pathways and biological properties of thyroid cancer, as detected in our RNA-seq results. The P53 signaling pathway, apoptosis, cellular senescence and PD-1 checkpoint pathway which have been validated to be regulated by KDM1A in different types of tumors 50-52 were also enriched in the KEGG pathway analysis of our RNA-seq. It's noticeable that the enrichment levels in the pathways of "platinum drug resistance", "EGFR tyrosine inhibitor resistance" and "ABC transporters" were down-regulated after KDM1A knockdown (Figure S3B), indicating that KDM1A may mediate the multidrug resistance (MDR) of ATC cells, which is thought to be a pivotal reason for the failure of chemotherapy in many cancers 53. These results require further confirmation and study but also indicate that KDM1A has multiple carcinogenic effects on thyroid cancer.

Moreover, an unignorable advantage of considering KDM1A as a therapeutic target is that many targeted inhibitors have been designed against it. Some of them have even undergone clinical trials and have achieved some curative effects 54. In recent years, new compounds targeting KDM1A have continuously emerged. TCP (Tranylcypromine) was the first identified KDM1A inhibitor that could suppress various cancers and most of the present KDM1A inhibitors are synthesized and modified based on TCP. GSK-LSD1, the KDM1A inhibitor used in this study also belongs to the TCP family. It can mimic KDM1A knockdown caused by shRNA both *in vitro* and *in vivo* and has shown favorable anticancer activity in varying cancers 24, 39, 43. Considering the good *in vivo* activity and low toxicity of GSK-LSD1, its application in the clinical treatment of advanced thyroid cancer is promising.

In summary, our study uncovers a novel regulatory mechanism of stemness and chemosensitivity in thyroid cancer and provides new insights into thyroid cancer therapy. The combination regimen of KDM1A targeted small molecule inhibitors and chemotherapy may provide an effective therapeutic strategy for patients with advanced thyroid cancer.

## Conclusion

In summary, the elevated KDM1A expression in thyroid cancer cells activates the Wnt/β-catenin signaling pathway through down-regulatingits two antagonists, APC2 and DKK1, by demethylating H3K4me1/2 of the APC2 promoter region and the nonhistone substrate HIF-1α, causing the inhibition of APC2 transcription and the activation of the HIF-1α/microRNA-146a/DKK1 axis. The KDM1A-activated canonical Wnt pathway leads to increased cancer stemness and attenuated chemosensitivity in thyroid cancer.

## Supplementary Material

Supplementary figures, tables, and additional files.

## Abbreviations

KDM1A: lysine demethylase 1A
PTC: papillary thyroid cancer
FTC: follicular thyroid carcinoma
PDTC: poorly differentiated thyroid carcinoma
ATC: anaplastic thyroid carcinoma
CSCs: cancer stem cells
Wnt: wingless/integrated
EMT: epithelial-to-mesenchymal transition
STR: short tandem repeat
IP: immunoprecipitation
shRNA: short hairpin RNA
siRNA: small interfering RNA
OCT4: organic cation/carnitine transporter 4
SOX2: SRY-box transcription factor 2
ALDH1: aldehyde dehydrogenase 1 family member A1
GO: Gene ontology
KEGG: Kyoto Encyclopedia of Genes and Genomes
GSEA: Gene Set Enrichment Analysis
APC2: APC regulator of WNT signaling pathway 2
DKK1: dickkopf WNT signaling pathway inhibitor 1
CCND1: cyclin D1
ChIP: chromatin immunoprecipitation
HIF-1α: hypoxia inducible factor 1 subunit alpha
UTR: untranslated region
MiRNA: microRNAs
WT: wild type
MUT: mutant
AKT: Akt serine/threonine kinase 1
MAPK: mitogen-activated protein kinase
GSK3β: glycogen synthase kinase 3β
TCP: Tranylcypromine

## References

[1] Siegel RL, Miller KD, Jemal A (2018). Cancer statistics, 2018. CA Cancer J Clin.

[2] Kitahara CM, Sosa JA (2016). The changing incidence of thyroid cancer. Nat Rev Endocrinol.

[3] Williams MD, Tischler AS (2017). Update from the 4th Edition of the World Health Organization Classification of Head and Neck Tumours: Paragangliomas. Head Neck Pathol.

[4] Cabanillas ME, McFadden DG, Durante C (2016). Thyroid cancer. Lancet.

[5] Siegel RL, Miller KD, Jemal A (2019). Cancer statistics, 2019. CA Cancer J Clin.

[6] Molinaro E, Romei C, Biagini A, Sabini E, Agate L, Mazzeo S (2017). Anaplastic thyroid carcinoma: from clinicopathology to genetics and advanced therapies. Nat Rev Endocrinol.

[7] Batlle E, Clevers H (2017). Cancer stem cells revisited. Nat Med.

[8] Tang T, Guo C, Xia T, Zhang R, Zen K, Pan Y (2019). LncCCAT1 Promotes Breast Cancer Stem Cell Function through Activating WNT/β-catenin Signaling. Theranostics.

[9] Visvader JE, Lindeman GJ (2012). Cancer stem cells: current status and evolving complexities. Cell Stem Cell.

[10] Lei ZJ, Wang J, Xiao HL, Guo Y, Wang T, Li Q (2015). Lysine-specific demethylase 1 promotes the stemness and chemoresistance of Lgr5 (+) liver cancer initiating cells by suppressing negative regulators of beta-catenin signaling. Oncogene.

[11] Antonelli A, La Motta C (2017). Novel therapeutic clues in thyroid carcinomas: The role of targeting cancer stem cells. Med Res Rev.

[12] Todaro M, Iovino F, Eterno V, Cammareri P, Gambara G, Espina V (2010). Tumorigenic and metastatic activity of human thyroid cancer stem cells. Cancer Res.

[13] Dawson MA, Kouzarides T (2012). Cancer epigenetics: from mechanism to therapy. Cell.

[14] Feinberg AP, Koldobskiy MA, Göndör A (2016). Epigenetic modulators, modifiers and mediators in cancer aetiology and progression. Nat Rev Genet.

[15] Song Y, Wu F, Wu J (2016). Targeting histone methylation for cancer therapy: enzymes, inhibitors, biological activity and perspectives. J Hematol Oncol.

[16] Shi Y, Lan F, Matson C, Mulligan P, Whetstine JR, Cole PA (2004). Histone demethylation mediated by the nuclear amine oxidase homolog LSD1. Cell.

[17] Metzger E, Wissmann M, Yin N, Müller JM, Schneider R, Peters AH (2005). LSD1 demethylates repressive histone marks to promote androgen-receptor-dependent transcription. Nature.

[18] Martin C, Zhang Y (2005). The diverse functions of histone lysine methylation. Nat Rev Mol Cell Biol.

[19] Lian Y, Yan C, Lian Y, Yang R, Chen Q, Ma D (2020). Long intergenic non-protein-coding RNA 01446 facilitates the proliferation and metastasis of gastric cancer cells through interacting with the histone lysine-specific demethylase LSD1. Cell Death Dis.

[20] Lin T, Ponn A, Hu X, Law BK, Lu J (2010). Requirement of the histone demethylase LSD1 in Snai1-mediated transcriptional repression during epithelial-mesenchymal transition. Oncogene.

[21] Ferrari-Amorotti G, Fragliasso V, Esteki R, Prudente Z, Soliera AR, Cattelani S (2013). Inhibiting interactions of lysine demethylase LSD1 with snail/slug blocks cancer cell invasion. Cancer Res.

[22] Yu Y, Schleich K, Yue B, Ji S, Lohneis P, Kemper K (2018). Targeting the Senescence-Overriding Cooperative Activity of Structurally Unrelated H3K9 Demethylases in Melanoma. Cancer Cell.

[23] Wu Y, Wang Y, Yang XH, Kang T, Zhao Y, Wang C (2013). The deubiquitinase USP28 stabilizes LSD1 and confers stem-cell-like traits to breast cancer cells. Cell Rep.

[24] Smitheman KN, Severson TM, Rajapurkar SR, McCabe MT, Karpinich N, Foley J (2019). Lysine specific demethylase 1 inactivation enhances differentiation and promotes cytotoxic response when combined with all-trans retinoic acid in acute myeloid leukemia across subtypes. Haematologica.

[25] Zhi J, Zhang P, Zhang W, Ruan X, Tian M, Guo S (2021). Inhibition of BRAF Sensitizes Thyroid Carcinoma to Immunotherapy by Enhancing tsMHCII-mediated Immune Recognition. J Clin Endocrinol Metab.

[26] Hou X, Shi X, Zhang W, Li D, Hu L, Yang J (2021). LDHA induces EMT gene transcription and regulates autophagy to promote the metastasis and tumorigenesis of papillary thyroid carcinoma. Cell Death Dis.

[27] Hosseini A, Minucci S (2017). A comprehensive review of lysine-specific demethylase 1 and its roles in cancer. Epigenomics.

[28] Long M, Zhu Y, Chen Z, Lin S, Peng X, Luo D (2020). Lysine-Specific Demethylase 1 Affects the Progression of Papillary Thyroid Carcinoma via HIF1α and microRNA-146a. J Clin Endocrinol Metab.

[29] Zhang W, Sun W, Qin Y, Wu C, He L, Zhang T (2019). Knockdown of KDM1A suppresses tumour migration and invasion by epigenetically regulating the TIMP1/MMP9 pathway in papillary thyroid cancer. J Cell Mol Med.

[30] Haddad RI, Lydiatt WM, Ball DW, Busaidy NL, Byrd D, Callender G (2015). Anaplastic Thyroid Carcinoma, Version 2.2015. J Natl Compr Canc Netw.

[31] Duchartre Y, Kim YM, Kahn M (2016). The Wnt signaling pathway in cancer. Crit Rev Oncol Hematol.

[32] Pronobis MI, Rusan NM, Peifer M (2015). A novel GSK3-regulated APC:Axin interaction regulates Wnt signaling by driving a catalytic cycle of efficient βcatenin destruction. Elife.

[33] Baetta R, Banfi C (2019). Dkk (Dickkopf) Proteins. Arterioscler Thromb Vasc Biol.

[34] Huang Z, Li S, Song W, Li X, Li Q, Zhang Z (2013). Lysine-specific demethylase 1 (LSD1/KDM1A) contributes to colorectal tumorigenesis via activation of the Wnt/beta-catenin pathway by down-regulating Dickkopf-1 (DKK1) [corrected]. PLoS One.

[35] Mazumdar J, O'Brien WT, Johnson RS, LaManna JC, Chavez JC, Klein PS (2010). O2 regulates stem cells through Wnt/β-catenin signalling. Nature Cell Biology.

[36] Lee JY, Park JH, Choi HJ, Won HY, Joo HS, Shin DH (2017). LSD1 demethylates HIF1α to inhibit hydroxylation and ubiquitin-mediated degradation in tumor angiogenesis. Oncogene.

[37] Di G, Kong L, Zhao Q, Ding T (2018). MicroRNA-146a knockdown suppresses the progression of ankylosing spondylitis by targeting dickkopf 1. Biomed Pharmacother.

[38] Li Y, Tao L, Zuo Z, Zhou Y, Qian X, Lin Y (2019). ZY0511, a novel, potent and selective LSD1 inhibitor, exhibits anticancer activity against solid tumors via the DDIT4/mTOR pathway. Cancer Lett.

[39] Mohammad HP, Smitheman KN, Kamat CD, Soong D, Federowicz KE, Van Aller GS (2015). A DNA Hypomethylation Signature Predicts Antitumor Activity of LSD1 Inhibitors in SCLC. Cancer Cell.

[40] Maes T, Mascaró C, Tirapu I, Estiarte A, Ciceri F, Lunardi S (2018). ORY-1001, a Potent and Selective Covalent KDM1A Inhibitor, for the Treatment of Acute Leukemia. Cancer Cell.

[41] Huang M, Chen C, Geng J, Han D, Wang T, Xie T (2017). Targeting KDM1A attenuates Wnt/beta-catenin signaling pathway to eliminate sorafenib-resistant stem-like cells in hepatocellular carcinoma. Cancer Lett.

[42] Bell CC, Fennell KA, Chan YC, Rambow F, Yeung MM, Vassiliadis D (2019). Targeting enhancer switching overcomes non-genetic drug resistance in acute myeloid leukaemia. Nat Commun.

[43] Verigos J, Karakaidos P, Kordias D, Papoudou-Bai A, Evangelou Z, Harissis HV (2019). The Histone Demethylase LSD1/KappaDM1A Mediates Chemoresistance in Breast Cancer via Regulation of a Stem Cell Program. Cancers (Basel).

[44] Wang J, Hevi S, Kurash JK, Lei H, Gay F, Bajko J (2009). The lysine demethylase LSD1 (KDM1) is required for maintenance of global DNA methylation. Nat Genet.

[45] Yang J, Huang J, Dasgupta M, Sears N, Miyagi M, Wang B (2010). Reversible methylation of promoter-bound STAT3 by histone-modifying enzymes. Proc Natl Acad Sci U S A.

[46] Xing M (2013). Molecular pathogenesis and mechanisms of thyroid cancer. Nat Rev Cancer.

[47] Saini S, Sripada L, Tulla K, Kumar P, Yue F, Kunda N (2019). Loss of MADD expression inhibits cellular growth and metastasis in anaplastic thyroid cancer. Cell Death Dis.

[48] Sastre-Perona A, Santisteban P (2012). Role of the wnt pathway in thyroid cancer. Front Endocrinol (Lausanne).

[49] Tartari CJ, Donadoni C, Manieri E, Mologni L, Mina PD, Villa A (2011). Dissection of the RET/β-catenin interaction in the TPC1 thyroid cancer cell line. Am J Cancer Res.

[50] Zhu L, Wang J, Kong W, Huang J, Dong B, Huang Y (2019). LSD1 inhibition suppresses the growth of clear cell renal cell carcinoma via upregulating P21 signaling. Acta Pharm Sin B.

[51] Zou ZK, Huang YQ, Zou Y, Zheng XK, Ma XD (2017). Silencing of LSD1 gene modulates histone methylation and acetylation and induces the apoptosis of JeKo-1 and MOLT-4 cells. Int J Mol Med.

[52] Qin Y, Vasilatos SN, Chen L, Wu H, Cao Z, Fu Y (2019). Inhibition of histone lysine-specific demethylase 1 elicits breast tumor immunity and enhances antitumor efficacy of immune checkpoint blockade. Oncogene.

[53] Bukowski K, Kciuk M, Kontek R (2020). Mechanisms of Multidrug Resistance in Cancer Chemotherapy. Int J Mol Sci.

[54] Fang Y, Liao G, Yu B (2019). LSD1/KDM1A inhibitors in clinical trials: advances and prospects. J Hematol Oncol.

