# Supplementary material for: KDM1A promotes thyroid cancer progression and maintains stemness through the Wnt/β-catenin signaling pathway
Source: Theranostics. 2022 Jan 3;12(4):1500–17. doi: 10.7150/thno.66142 (PMC8825597; doi:10.7150/thno.66142)
Supplement: Supplementary file 1 — Supplementary figures, tables, and additional files. [file thnov12p1500s1.pdf]

Supplementary Files

KDM1A promotes thyroid cancer progression and maintains stemness through the Wnt/β-catenin signaling pathway

Wei Zhang, Xianhui Ruan, Yaoshuang Li, Jingtai Zhi, Linfei Hu, Xiukun Hou, Xianle Shi, Xin Wang, Jinpeng Wang, Weike Ma, Pengfei Gu, Xiangqian Zheng and Ming Gao

Supplementary figure legends and supplementary figures

Figure S1

Figure S2

Figure S3

Figure S4

Figure S5

Figure S6

Figure S7

Figure S8

Figure S9

Figure S10

Figure S11

Supplementary tables

Table S1

Table S2

Table S3

Additional file 1

Additional file 2

Supplementary figure legends and supplementary figures

Figure S1

A. CSCs (spheres) derived from three thyroid cancer cell lines have higher expression of stemness-associated genes than their corresponding non-CSCs (monolayer cells).

B. Phylogenetic analysis of the histone methylation modifiers included in this study.

C. The relative expression levels of KDM1A were assessed by qPCR in 21 paired normal thyroid tissues and PTC tissues, relative KDM1A expression data was normalized with β-actin and represented on a log2 scale.

D. Comparison of the KDM1A mRNA levels between all normal thyroid tissues and PTC tissues derived from the TCGA database.

E. Comparison of the KDM1A mRNA levels between paired normal thyroid tissues and PTC tissues derived from the TCGA database.

F. The protein expression levels of KDM1A were assessed by Western blotting in a cohort of thyroid cancer cell lines and a normal thyroid cell line.

G. The relative expression levels of KDM1A were measured by qPCR in a cohort of thyroid cancer cell lines and a normal thyroid cell line.

Data are shown as the mean ± SD of three replicates. (*$P < 0.05$, **$P < 0.01$, ***$P < 0.001$)

Figure S2

A, B. Analysis of the levels of H3K4me1, H3K4me2, H3K4me3, H3K9me1, H3K9me2 and H3K9me3 in ATC cells following KDM1A knockdown (A) and in PTC cells following KDM1A overexpression (B) by western blotting.

C, D. The relative expression levels of some CSC markers were measured by qPCR in shKDM1A-transfected ATC cells (C) and KDM1A overexpression plasmid-transfected PTC cells (D).

Data are shown as the mean ± SD of three replicates. (*$P < 0.05$, **$P < 0.01$, ***$P < 0.001$)

Figure S3

A, B. The cell migration and invasion abilities of ATC cells with KDM1A knockdown (A) and PTC cells with KDM1A overexpression (B). The scale bar is 200 μm.

Data are shown as the mean ± SD of three replicates. (**$P < 0.01$, ***$P < 0.001$)

Figure S4

A. Cell viability of KDM1A overexpression plasmid-transfected PTC cells after treatment with doxorubicin at different concentration gradients for 48 h.

B. Flow cytometry was performed to measure the apoptotic proportion of KDM1A-silenced ATC cells after treatment with doxorubicin at a concentration of 100 nmol/L for 3 days.

C. Cell viability of shKDM1A-transfected ATCs after treatment with sorafenib at different concentrations for 48 h.

Data are shown as the mean ± SD of three replicates. (** $P < 0.01$, *** $P < 0.001$)

Figure S5

A. Representative GO term analysis of upregulated genes (red) and downregulated genes (blue) after transfection with shKDM1A.

B. Quantitative PCR validation of differential gene expression related to the Wnt/β-catenin signaling pathway in ATC cells with KDM1A knockdown.

E. The expression levels of β-catenin, DKK1 and APC2 in xenografts of each group were assessed by immunohistochemical staining. Unpaired two-tailed Student's *t*-test was used to analyzed the difference of positive staining cells ratio between different groups. The scale bar is 50 μm.

Data are shown as the mean ± SD of three replicates. (** $P < 0.01$, *** $P < 0.001$)

Figure S6

A. A TOP/FOP luciferase reporter assay was utilized to detect the transcriptional activity of the canonical Wnt signaling pathway in PTC cells with KDM1A overexpression.

B, C. Western blotting (B) and qPCR (C) validation of differential gene expression related to the Wnt/β-catenin signaling pathway in PTC cells with KDM1A overexpression.

D. Immunofluorescence of PTC cells with or without KDM1A overexpression for β-catenin (orange) and 4′,6-diamidino-2-phenylindole (DAPI) (blue). The scale bar is 20 μm.

Data are shown as the mean ± SD of three replicates. (** $P < 0.01$, *** $P < 0.001$)

Figure S7

A. Schematic of the ChIP primers designed for the DKK1 promoter regions (1–7).

B, C, D. The enrichment levels of KDM1A (B), H3K4me1 (C) and H3K4me2 (D) in different regions of the DKK1 gene promoter in CAL-62 ATC cells with or without KDM1A knockdown were determined by ChIP assay.

Data are shown as the mean ± SD of three replicates.

Figure S8

A. The protein expression of HIF-1α was examined by Western blotting in PTC cells with KDM1A overexpression.

B, C. The relative mRNA expression of HIF-1α was measured by qPCR in shKDM1A-transfected ATCs (B) and PTC cells with KDM1A overexpression.

D, E. Introduction of HIF-1α to ATC cells rescued their attenuated sphere formation ability (D) and increased their sensitivity to doxorubicin (E) caused by KDM1A knockdown.

F. Overexpression of HIF-1α in ATC cells with KDM1A knockdown restored the increased mRNA

expression of DKK1.

Data are shown as the mean ± SD of three replicates. (*$P < 0.05$, **$P < 0.01$, ***$P < 0.001$)

Figure S9

A. The relative expression levels of miR-146a were measured by qPCR in KDM1A overexpression plasmid-transfected PTC cells.

B, C. Introduction of miR-146a mimic to ATC cells restored their attenuated sphere formation ability (B) and increased their response to doxorubicin (C) caused by KDM1A overexpression.

D. Western blotting validated that the introduction of a miR-146a inhibitor to PTC cells could restore the increased β-catenin and CSC marker expression induced by KDM1A overexpression.

E. Quantitative PCR validated that the introduction of a miR-146a inhibitor to PTC cells could restore the decreased DKK1 expression induced by KDM1A knockdown.

F, G. Introduction of a miR-146a inhibitor to PTC cells restored the enhanced sphere formation ability (F) and the resistance to doxorubicin (G) caused by KDM1A overexpression.

Data are shown as the mean ± SD of three replicates. (**$P < 0.01$, ***$P < 0.001$)

Figure S10

A. Flow cytometry apoptosis analysis confirmed the efficacy of combination therapy in which ATCs were pretreated with 1 μM GSK-LSD1 for 5 days and then subjected to 100 nmol/L doxorubicin treatment for another 3 days.

B. The cell migration and invasion abilities of ATC cells that had been treated with GSK-LSD1 (1 μM) for 5 days. The scale bar is 200 μm.

Data are shown as the mean ± SD of three replicates. (**$P < 0.01$, ***$P < 0.001$)

Figure S11

A. The alteration in body weight in each group of mice after treatment.

B. The expression levels of Ki67, cleaved caspase 3, NANOG, β-catenin, APC2 and DKK1 in xenografts of each group were assessed by immunohistochemical staining. Unpaired two-tailed Student's *t*-test was used to analyzed the difference of positive staining cells ratio between different groups. The scale bar is 50 μm.

Data are shown as the mean ± SD of three replicates. (*$P < 0.05$, ***$P < 0.001$)

Figure S1

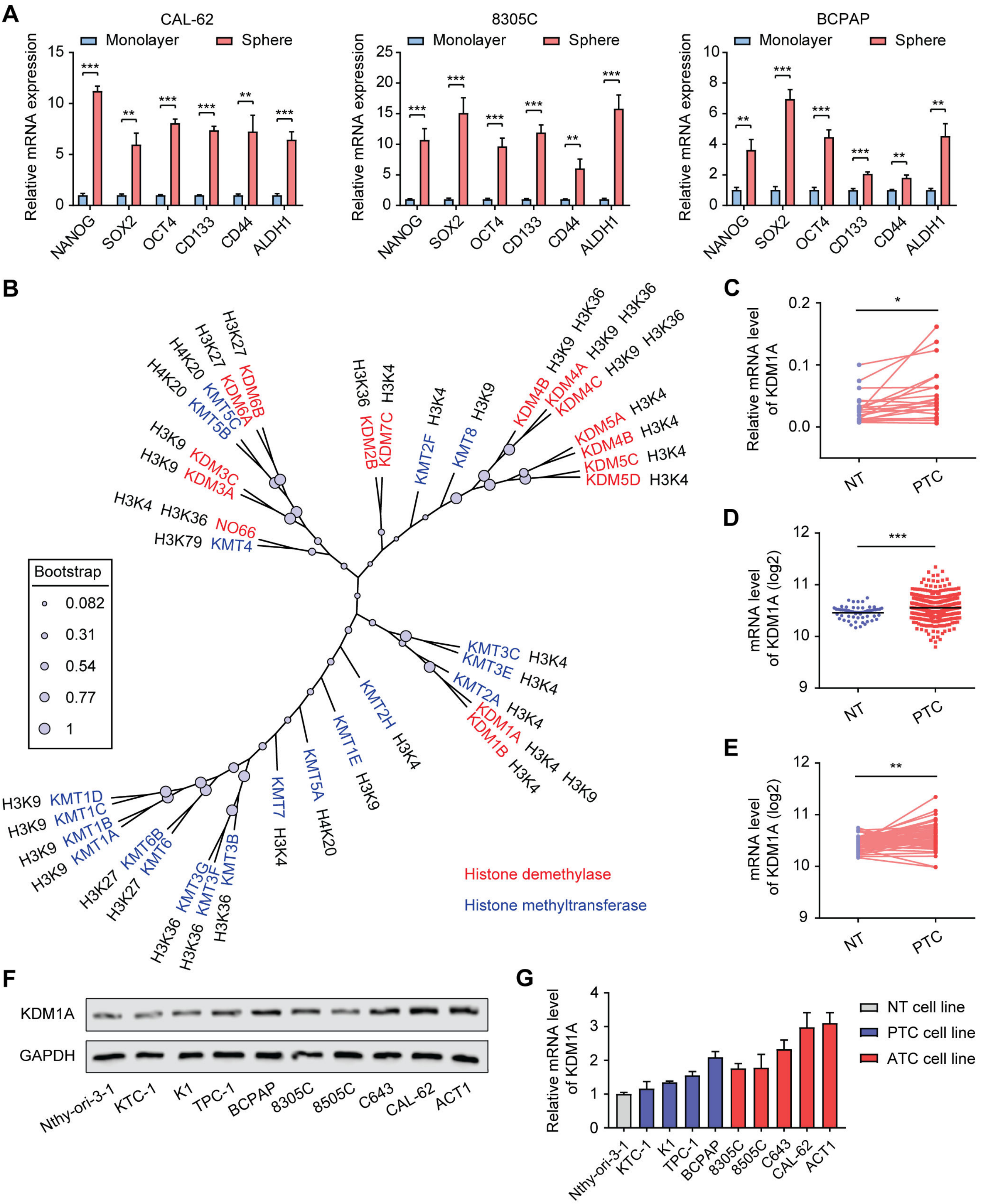

A
CAL-62
8305C
BCPAP
Monolayer
Sphere
Relative mRNA expression
NANOG
SOX2
OCT4
CD133
CD44
ALDH1
B
Bootstrap
0.082
0.31
0.54
0.77
1
Histone demethylase
Histone methyltransferase
C
Relative mRNA level of KDM1A
D
mRNA level of KDM1A (log2)
E
NT
PTC
F
KDM1A
GAPDH
Nthy-ori-3-1
KTC-1
K1
TPC-1
BCPAP
8305C
8505C
C643
CAL-62
ACT1
G
NT cell line
PTC cell line
ATC cell line

Figure S2

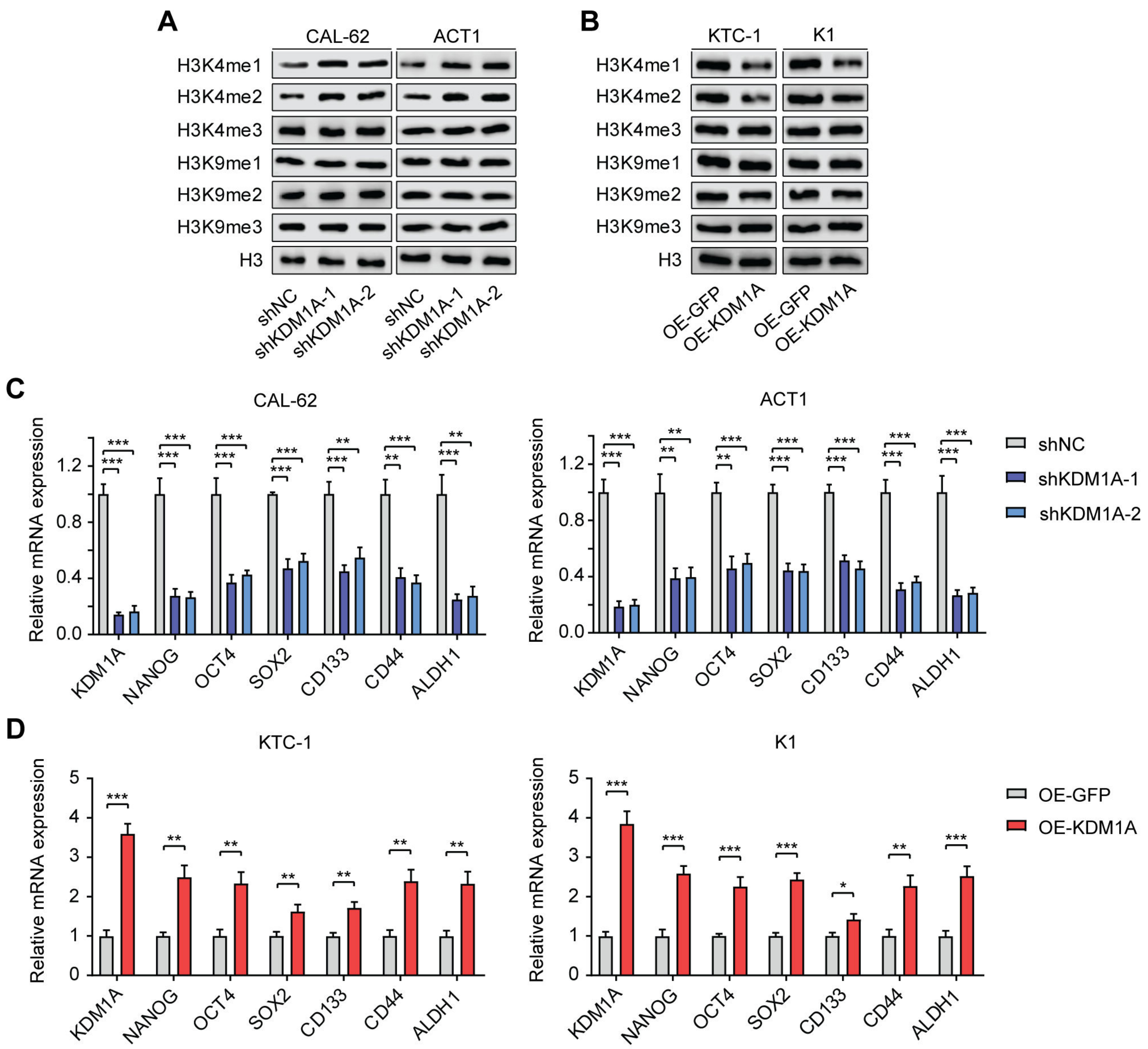
A
CAL-62
ACT1
H3K4me1
H3K4me2
H3K4me3
H3K9me1
H3K9me2
H3K9me3
H3
shNC
shKDM1A-1
shKDM1A-2
B
KTC-1
K1
OE-GFP
OE-KDM1A
C
CAL-62
ACT1
Relative mRNA expression
KDM1A
NANOG
OCT4
SOX2
CD133
CD44
ALDH1
shNC
shKDM1A-1
shKDM1A-2
D
KTC-1
K1
OE-GFP
OE-KDM1A

Figure S3

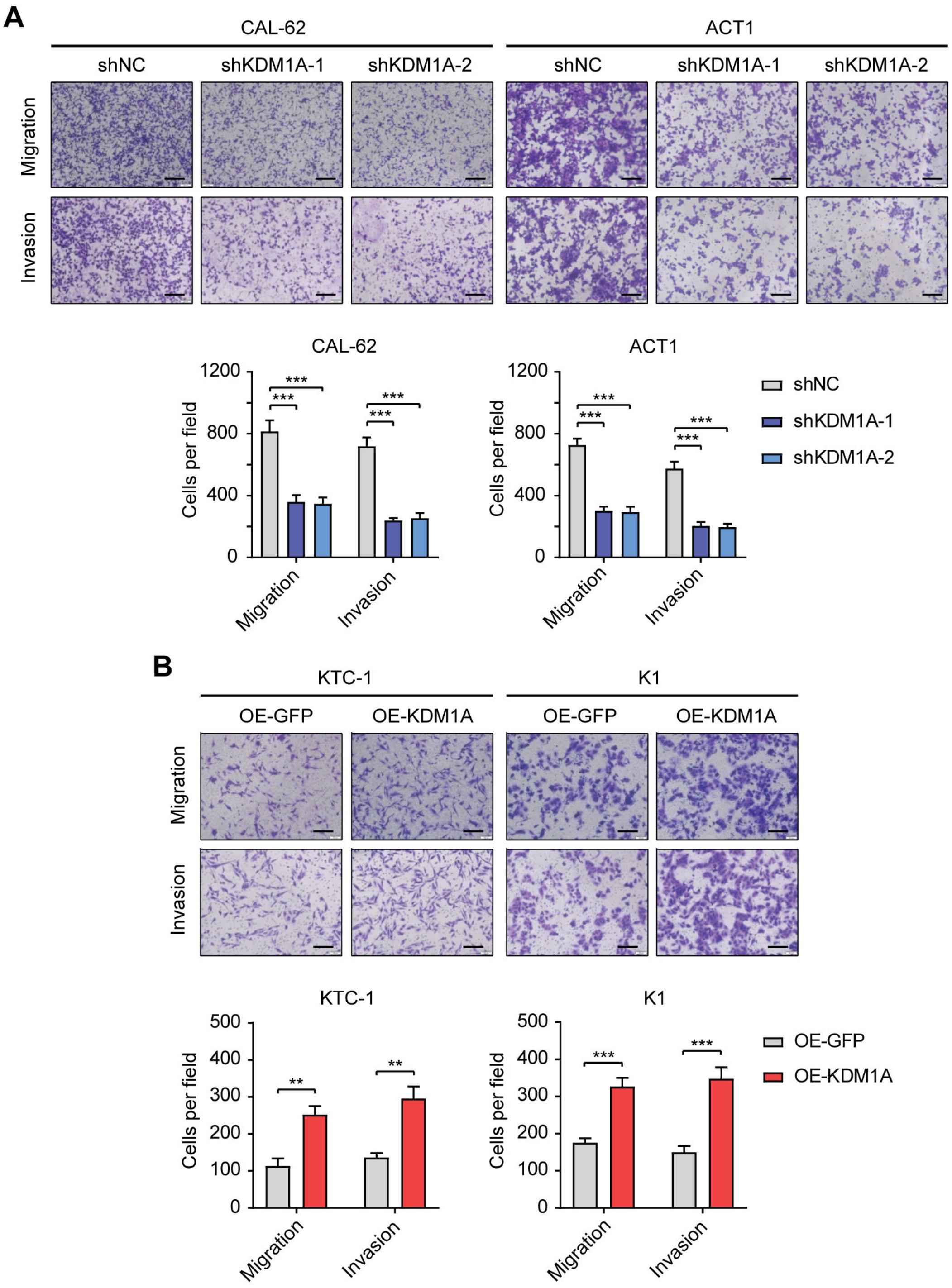
A
CAL-62
ACT1
shNC
shKDM1A-1
shKDM1A-2
Migration
Invasion
Cells per field
1200
800
400
0
***
**
shNC
shKDM1A-1
shKDM1A-2
B
KTC-1
K1
OE-GFP
OE-KDM1A
500
400
300
200
100

Figure S4

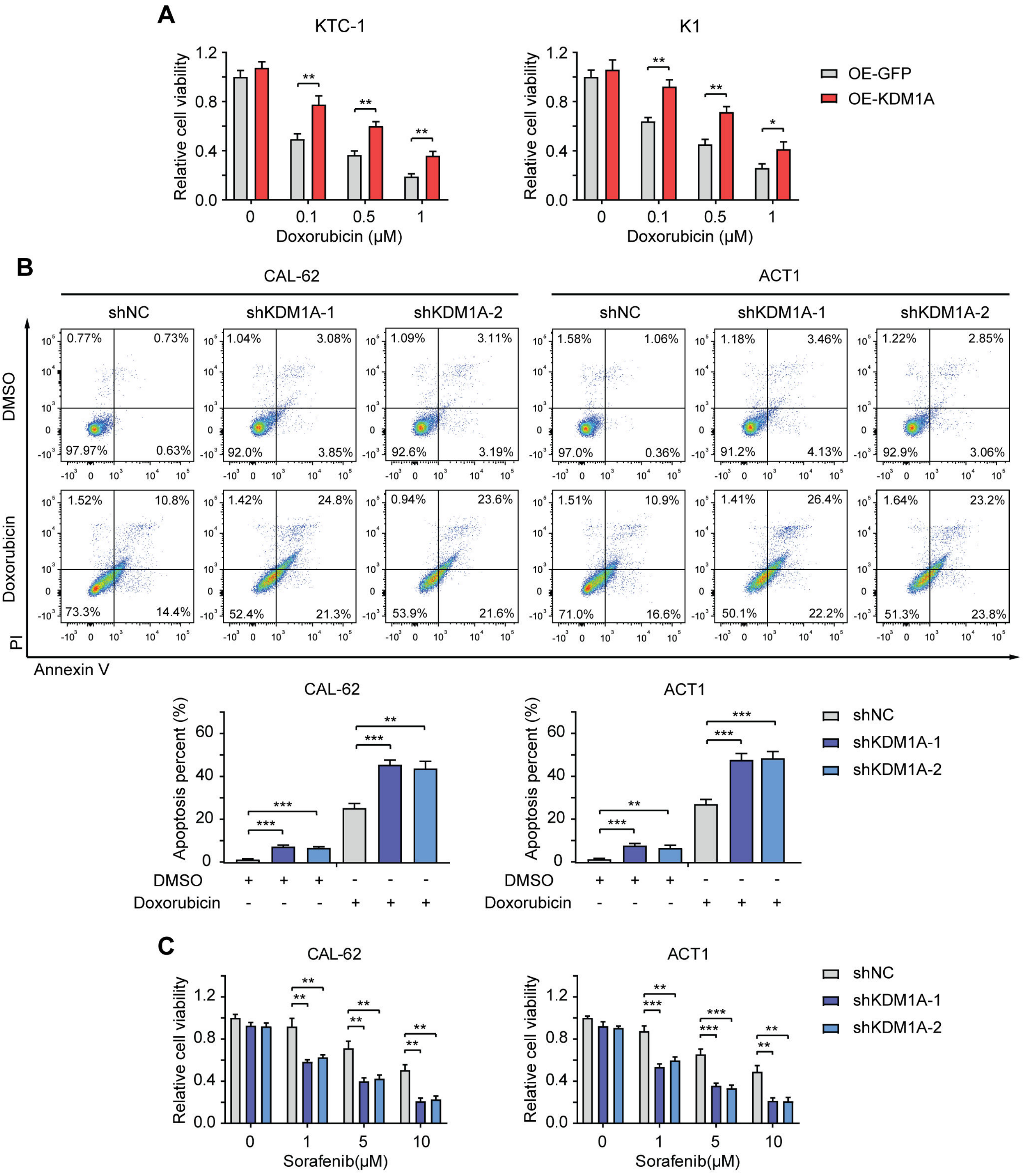

A
KTC-1
K1
Relative cell viability
Doxorubicin (μM)
OE-GFP
OE-KDM1A
B
CAL-62
ACT1
shNC
shKDM1A-1
shKDM1A-2
DMSO
Doxorubicin
PI
Annexin V
0.77%
0.73%
97.97%
0.63%
1.04%
3.08%
92.0%
3.85%
1.09%
3.11%
92.6%
3.19%
1.58%
1.06%
97.0%
0.36%
1.18%
3.46%
91.2%
4.13%
1.22%
2.85%
92.9%
3.06%
1.52%
10.8%
73.3%
14.4%
1.42%
24.8%
52.4%
21.3%
0.94%
23.6%
53.9%
21.6%
1.51%
10.9%
71.0%
16.6%
1.41%
26.4%
50.1%
22.2%
1.64%
23.2%
51.3%
23.8%
Apoptosis percent (%)
C
Sorafenib(μM)

Figure S5

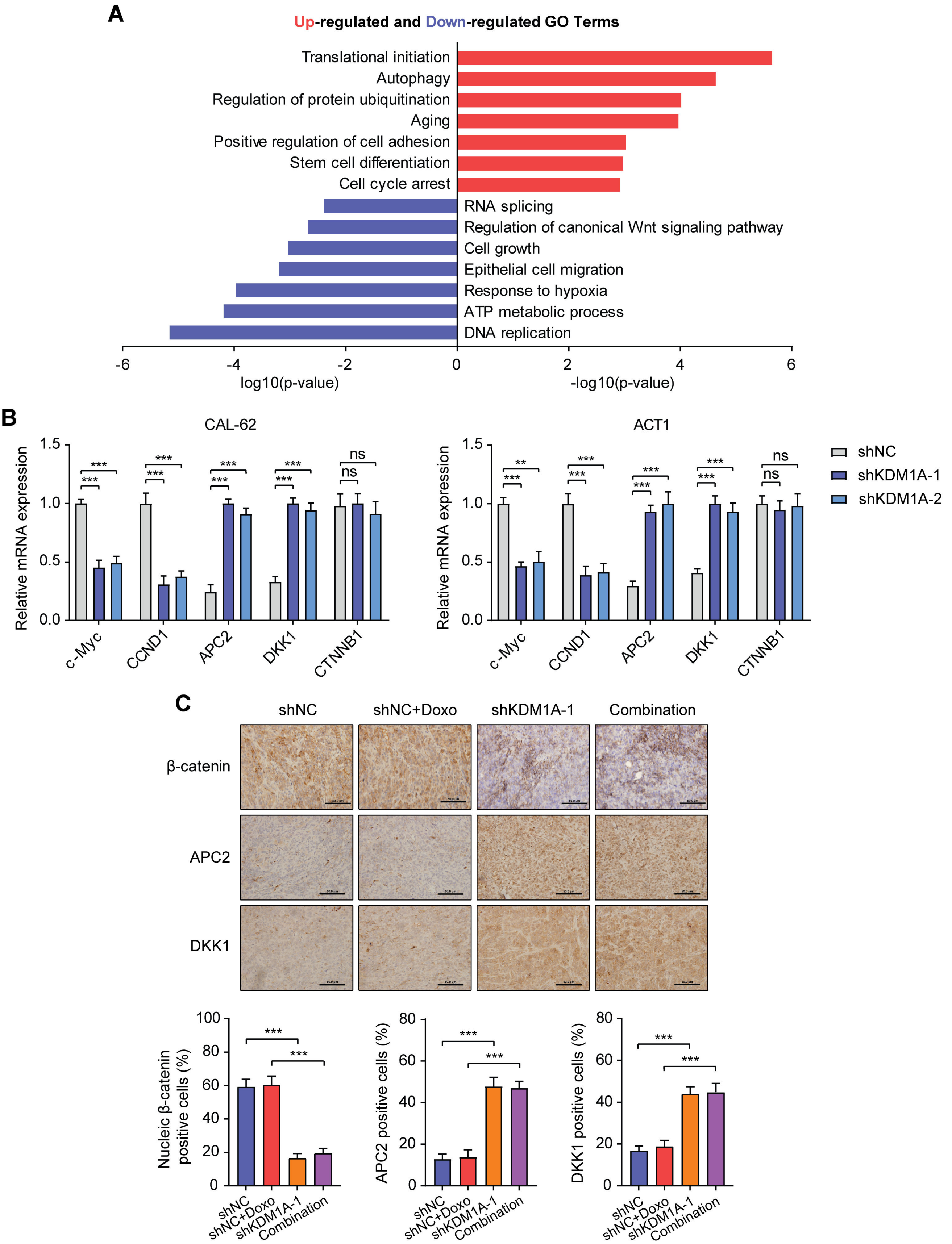

A
Up-regulated and Down-regulated GO Terms
Translational initiation
Autophagy
Regulation of protein ubiquitination
Aging
Positive regulation of cell adhesion
Stem cell differentiation
Cell cycle arrest
RNA splicing
Regulation of canonical Wnt signaling pathway
Cell growth
Epithelial cell migration
Response to hypoxia
ATP metabolic process
DNA replication
log10(p-value)
-log10(p-value)
B
CAL-62
ACT1
Relative mRNA expression
c-Myc
CCND1
APC2
DKK1
CTNNB1
shNC
shKDM1A-1
shKDM1A-2
ns
C
shNC
shNC+Doxo
shKDM1A-1
Combination
β-catenin
APC2
DKK1
Nucleic β-catenin positive cells (%)
APC2 positive cells (%)
DKK1 positive cells (%)

Figure S6

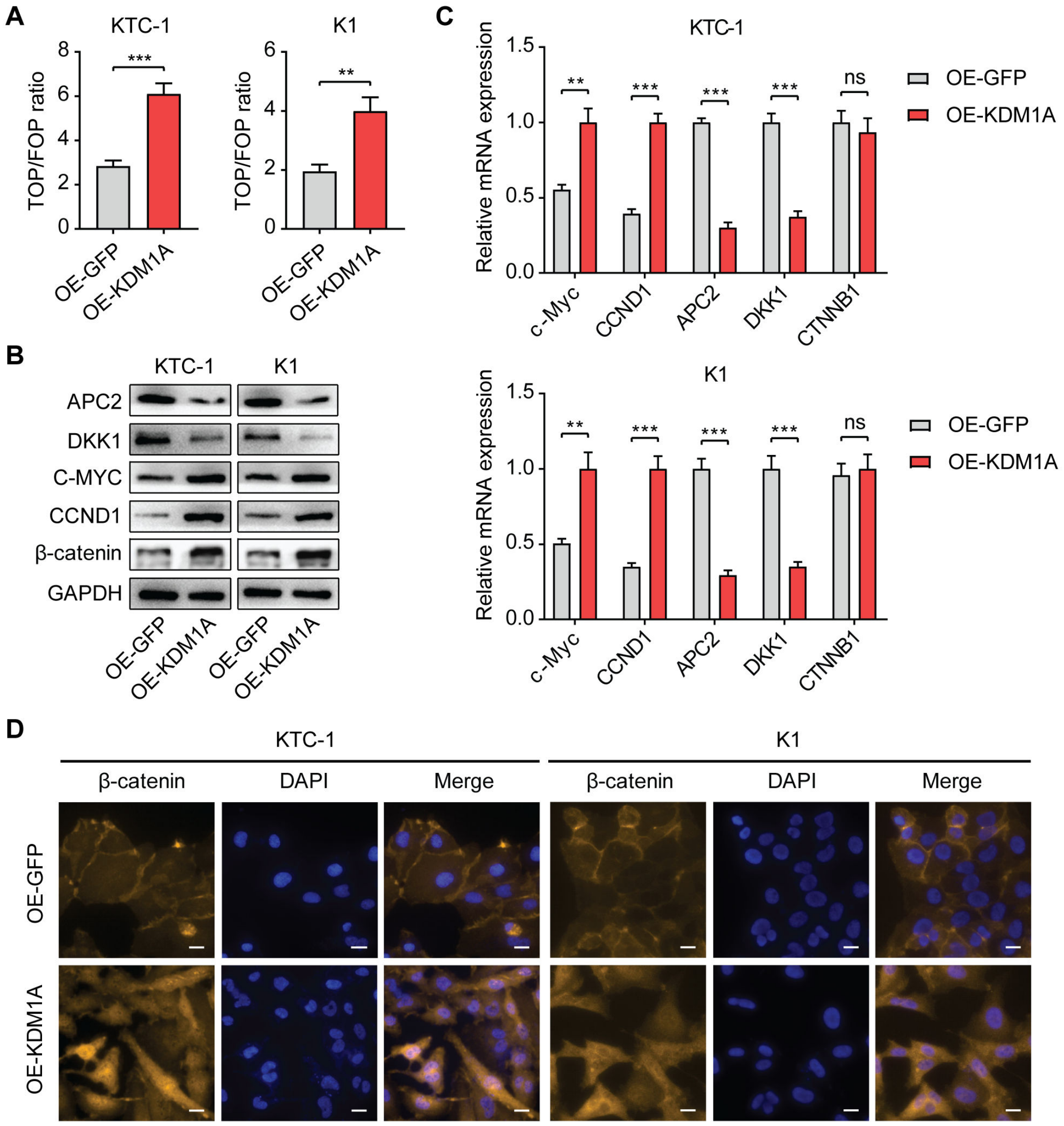

A
KTC-1
K1
TOP/FOP ratio
***
**
OE-GFP
OE-KDM1A
B
KTC-1
K1
APC2
DKK1
C-MYC
CCND1
β-catenin
GAPDH
OE-GFP
OE-KDM1A
C
KTC-1
K1
Relative mRNA expression
**
***
ns
c-Myc
CCND1
APC2
DKK1
CTNNB1
OE-GFP
OE-KDM1A
D
KTC-1
K1
β-catenin
DAPI
Merge
OE-GFP
OE-KDM1A

Figure S7

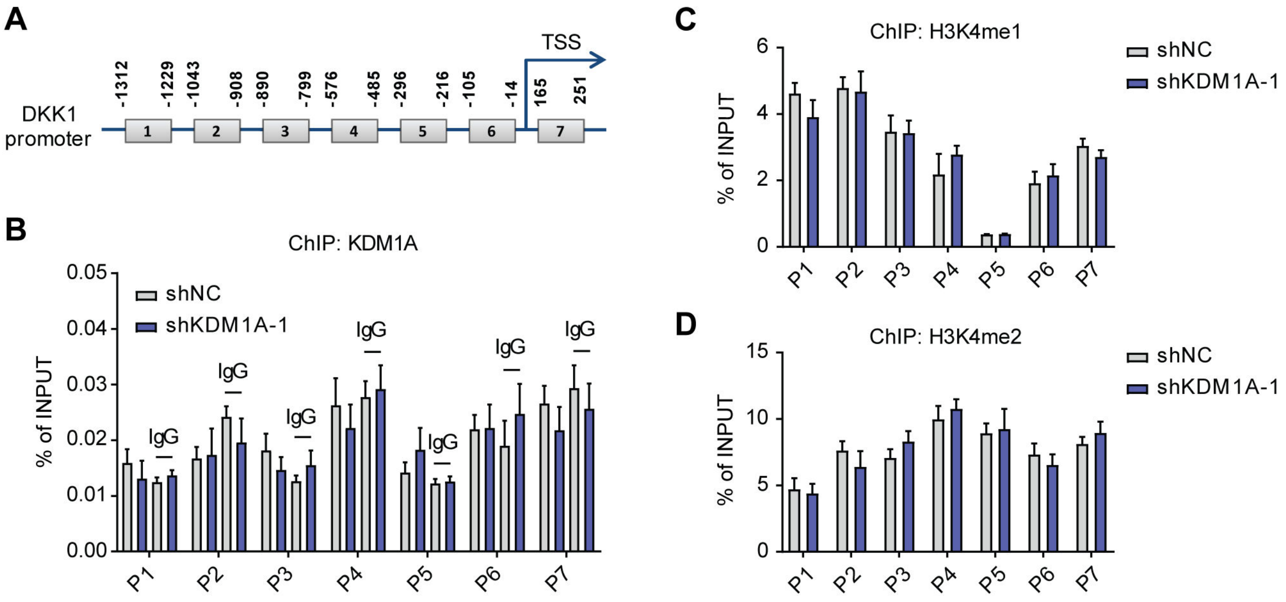

A
TSS
-1312
-1229
-1043
-908
-890
-799
-576
-485
-296
-216
-105
-14
165
251
DKK1 promoter
1
2
3
4
5
6
7
B
ChIP: KDM1A
% of INPUT
0.05
0.04
0.03
0.02
0.01
0.00
shNC
shKDM1A-1
IgG
P1
P2
P3
P4
P5
P6
P7
C
ChIP: H3K4me1
% of INPUT
6
4
2
0
shNC
shKDM1A-1
P1
P2
P3
P4
P5
P6
P7
D
ChIP: H3K4me2
% of INPUT
15
10
5
0
shNC
shKDM1A-1
P1
P2
P3
P4
P5
P6
P7

Figure S8

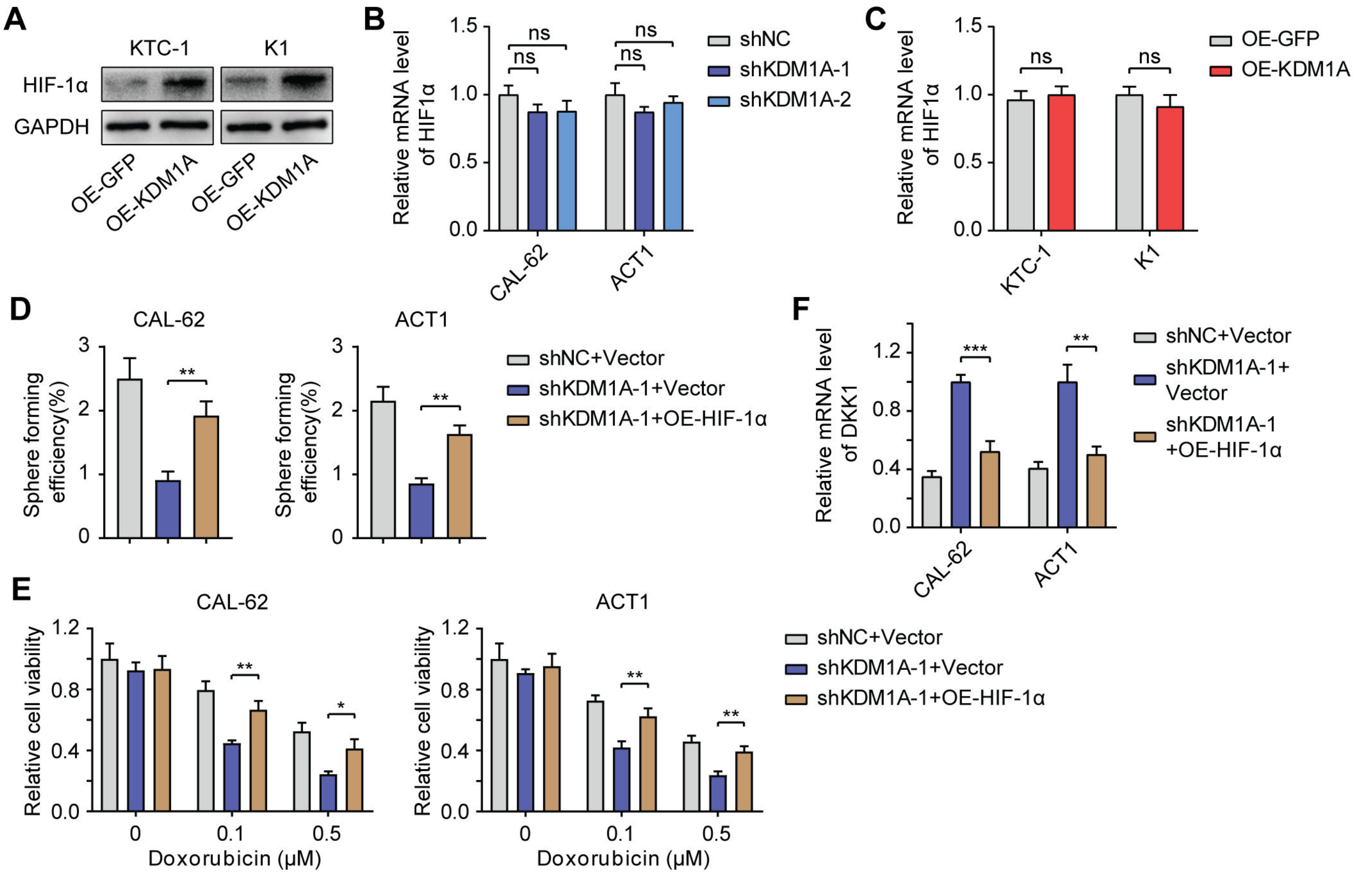

A
KTC-1
K1
HIF-1α
GAPDH
OE-GFP
OE-KDM1A
OE-GFP
OE-KDM1A
B
Relative mRNA level of HIF1α
ns
ns
ns
ns
CAL-62
ACT1
shNC
shKDM1A-1
shKDM1A-2
C
Relative mRNA level of HIF1α
ns
ns
KTC-1
K1
OE-GFP
OE-KDM1A
D
CAL-62
ACT1
Sphere forming efficiency(%)
**
**
shNC+Vector
shKDM1A-1+Vector
shKDM1A-1+OE-HIF-1α
F
Relative mRNA level of DKK1
***
**
CAL-62
ACT1
shNC+Vector
shKDM1A-1+ Vector
shKDM1A-1 +OE-HIF-1α
E
CAL-62
ACT1
Relative cell viability
Doxorubicin (μM)
**
*
**
**
shNC+Vector
shKDM1A-1+Vector
shKDM1A-1+OE-HIF-1α

Figure S9

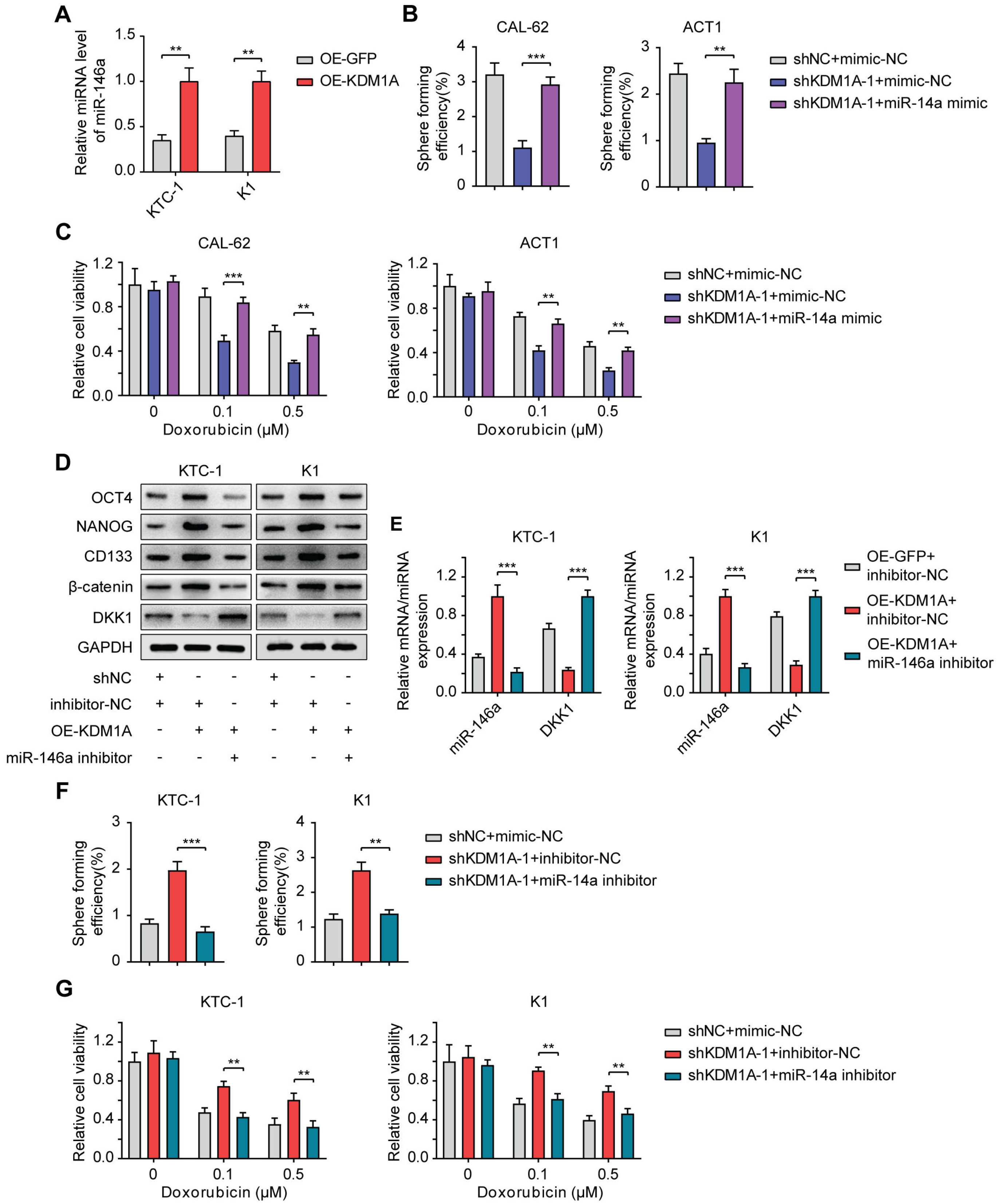
A
Relative miRNA level of miR-146a
1.5
1.0
0.5
0.0
**
KTC-1
K1
OE-GFP
OE-KDM1A
B
CAL-62
ACT1
Sphere forming efficiency(%)
***
shNC+mimic-NC
shKDM1A-1+mimic-NC
shKDM1A-1+miR-14a mimic
C
CAL-62
ACT1
Relative cell viability
1.2
0.8
0.4
0.0
0
0.1
0.5
Doxorubicin (μM)
shNC+mimic-NC
shKDM1A-1+mimic-NC
shKDM1A-1+miR-14a mimic
D
KTC-1
K1
OCT4
NANOG
CD133
β-catenin
DKK1
GAPDH
shNC
inhibitor-NC
OE-KDM1A
miR-146a inhibitor
E
KTC-1
K1
Relative mRNA/miRNA expression
miR-146a
DKK1
OE-GFP+ inhibitor-NC
OE-KDM1A+ inhibitor-NC
OE-KDM1A+ miR-146a inhibitor
F
KTC-1
K1
Sphere forming efficiency(%)
shNC+mimic-NC
shKDM1A-1+inhibitor-NC
shKDM1A-1+miR-14a inhibitor
G
KTC-1
K1
Relative cell viability
Doxorubicin (μM)
shNC+mimic-NC
shKDM1A-1+inhibitor-NC
shKDM1A-1+miR-14a inhibitor

Figure S10

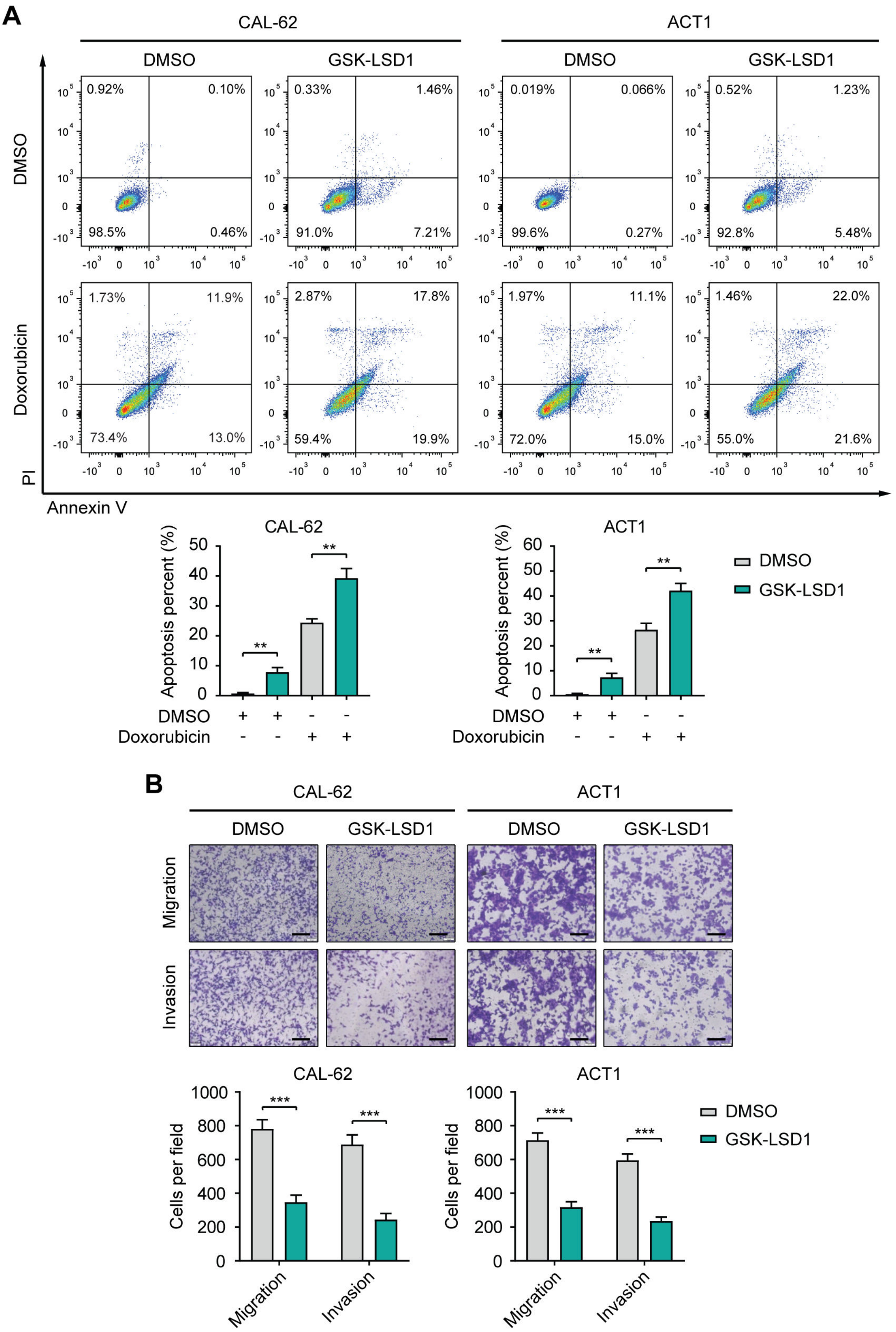

A
CAL-62
ACT1
DMSO
GSK-LSD1
DMSO
Doxorubicin
PI
Annexin V
0.92%
0.10%
98.5%
0.46%
0.33%
1.46%
91.0%
7.21%
0.019%
0.066%
99.6%
0.27%
0.52%
1.23%
92.8%
5.48%
1.73%
11.9%
73.4%
13.0%
2.87%
17.8%
59.4%
19.9%
1.97%
11.1%
72.0%
15.0%
1.46%
22.0%
55.0%
21.6%
Apoptosis percent (%)
DMSO
Doxorubicin
B
Migration
Invasion
Cells per field

Figure S11

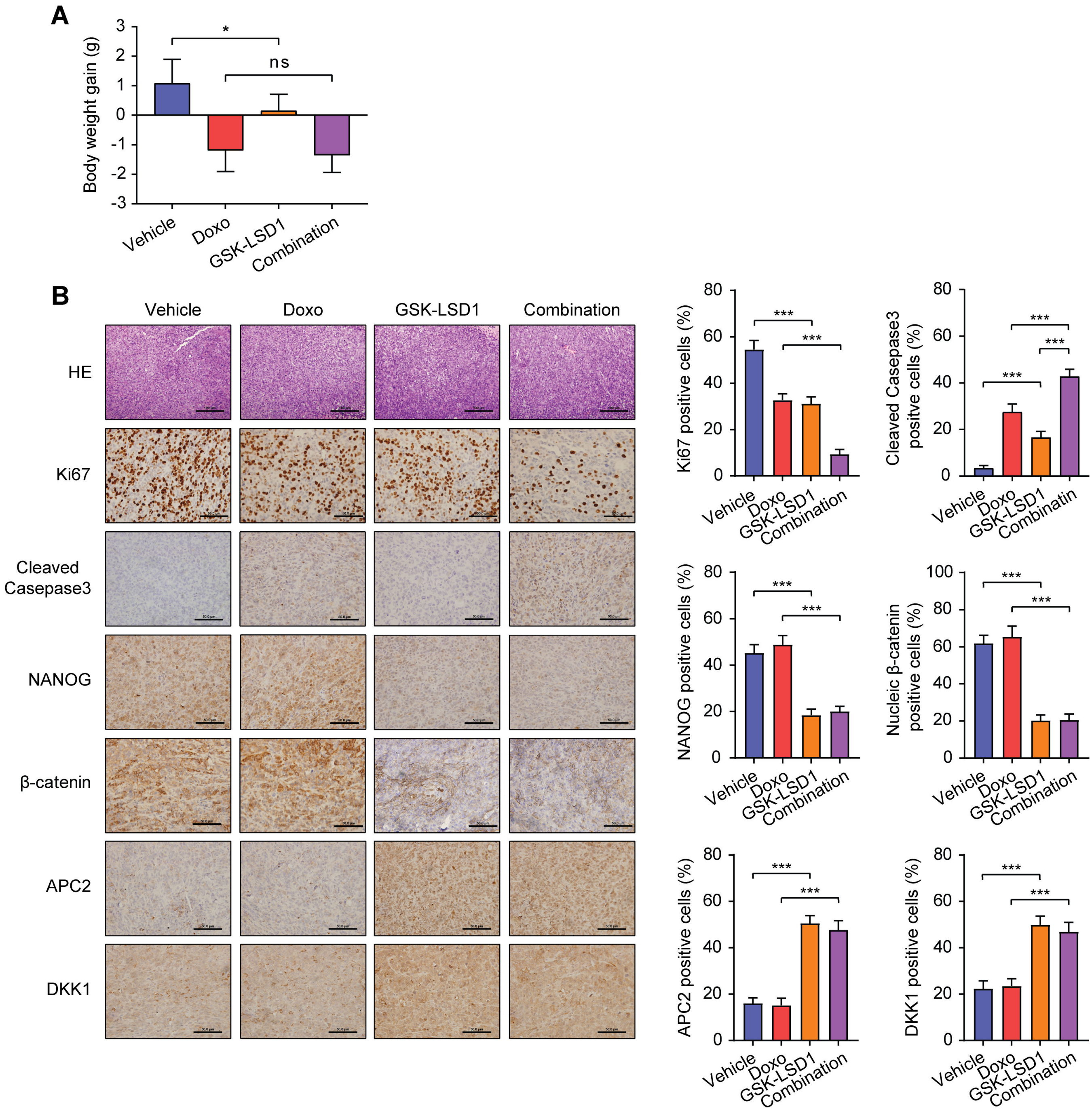
A
Body weight gain (g)
3
2
1
0
-1
-2
-3
*
ns
Vehicle
Doxo
GSK-LSD1
Combination
B
Vehicle
Doxo
GSK-LSD1
Combination
HE
Ki67
Cleaved Casepase3
NANOG
β-catenin
APC2
DKK1
Ki67 positive cells (%)
Cleaved Casepase3 positve cells (%)
NANOG positive cells (%)
Nucleic β-catenin positive cells (%)
APC2 positive cells (%)
DKK1 positive cells (%)
***
Combinatin
0
20
40
60
80
100

Supplementary tables

Table S1. The fold change (log2) of histone methylation modifiers expression in CSCs and no-CSCs (CSCs/no-CSCs)

Gene Name	BCPAP	8305C	CAL-62	Average fold change
KMT6	-0.55967	-0.379	-0.007	-0.315223333
KMT3G	0.371	-0.96733	-0.18833	-0.261553333
KMT3E	-0.31667	-0.23933	-0.05667	-0.204223333
KMT1B	-0.12133	-0.21267	0.445333	0.037111
KMT7	1.389667	-0.74233	-0.51867	0.042889
NO66	0.049333	0.008333	0.386667	0.148111
KMT3C	-0.00933	0.151	0.312667	0.151445667
KDM5D	0.27382	0.475784	0.047667	0.265757
KMT5B	0.083333	0.398333	0.36	0.280555333
KMT5A	0.322667	0.174	0.39	0.295555667
KDM4A	1.976667	-0.65267	0.226	0.516665667
KMT3F	0.722667	0.325333	0.555333	0.534444333
KMT1A	1.266	0.399333	-0.04267	0.540887667
KMT4	1.448	0.046667	0.184	0.559555667
KMT6B	1.013	0.246667	0.554	0.604555667
KMT1E	0.811667	0.010667	1.087	0.636444667
KDM7C	1.195667	0.314667	0.639667	0.716667
KDM6A	1.071	0.042667	1.040333	0.718
KMT5C	1.375	0.501333	0.294	0.723444333
KDM3A	1.235667	0.888667	0.408333	0.844222333
KMT1D	1.688667	0.533667	0.311667	0.844667
KDM4C	1.412333	0.314667	1.202333	0.976444333
KMT2H	2.193	0.693333	0.259333	1.048555333
KMT1C	2.597667	1.094667	-0.14967	1.180888
KDM3C	1.189	1.129	1.37	1.229333333
KMT3B	1.509667	1.880333	0.33	1.24
KDM4B	1.966333	0.908333	0.846	1.240222
KMT2A	1.850333	1.373	0.662	1.295111
KDM2B	1.310667	2.441	0.244667	1.332111333
KMT2	2.014333	0.226667	1.831333	1.357444333

KMT8	2.24	1.154667	0.713667	1.369444667
KDM5C	2.117	3.112333	0.758667	1.996
KDM1B	1.353333	1.243667	3.426667	2.007889
KDM5A	2.039	1.307	3.340667	2.228889
KDM6B	2.828	2.959333	1.579333	2.455555333
KDM1A	1.702667	2.882833	2.921667	2.502389
KDM5B	2.368667	3.980333	3.284	3.211

Table S2. Analysis of KDM1A expression and Clinicopathological features in PTC

Characteristic	n	KMD1A		χ^2	P-value
	(136)	+	-		
Age, y					
< 55	26	64	46	2.142	0.143
≧ 55	110	11	15		
Gender					
Male	39	20	19	0.330	0.566
Female	97	55	2		
Multifocality					
No	101	55	46	0.076	0.783
Yes	35	20	15		
BRAF mutation					
Yes	67	43	24	4.355	0.037
No	69	32	37		
Tumor size					
< 2cm	112	61	51	0.120	0.729
≧ 2cm	24	14	10		
Lymph node metastasis					
Yes	78	50	28	4.354	0.015
No	58	25	33		
TNM stage					
I-II	116	67	49	2.175	0.140
III-IV	20	8	12		

Table S3. Multivariate analysis of lymph node metastasis and clinicopathological features in PTC

Characteristic	B	S.E.	Wals	df	P	OR	95% C.I.	
							Lower	Upper
Age	-0.060	0.026	5.399	1	0.020	0.942	0.895	0.991
Gender	-0.752	0.451	2.774	1	0.096	0.472	0.195	1.142
Multifocal-ity	0269	0.453	0.351	1	0.553	1.308	0.538	3.183
$BRAF^{V600E}$ mutation	-1.399	0.420	11.070	1	0.001	0.247	0.108	0.563
Tumor size	-0.916	0.534	2.940	1	0.086	0.400	0.140	1.140
KDM1A expression	1.202	0.426	7.971	1	0.005	3.328	1.444	7.667
Constant	4.000	1.428	7.850	1	0.005	54.625		

Additional file 1

ALDEFLUOR assay

Cells were analyzed by an ALDEFLUOR assay kit (Stemcell Technologies; Vancouver, BC, Canada) according to the manufacturer's protocol. Flow cytometry was performed on a FACS Calibur flow cytometer (BD Biosciences) and the data were analyzed by FlowJo software (version 10.0.7; FlowJo LLC, OR).

Cell viability assay

Cells were seeded in 96-well culture plates at 2000 cells/well. After 12 h, cells were treated with drugs. After 48 h of treatment, cell viability was detected using a Cell Counting Kit-8 (SolarBio) according to the manufacturer's instructions.

***In vivo* studies**

All animal studies were approved by the Ethics Committee of the Tianjin Medical University Cancer Institute and Hospital. For the limiting dilution assay, varying amounts of CAL-62 cells with or without KDM1A knockdown were subcutaneously injected into 6-week-old female BALB/c nude mice. 6 weeks later, the number of mice that had developed tumors was counted. The frequency of CSCs was calculated using ELDA software. To establish a xenograft tumor model, 2×10^6 cells were subcutaneously injected into 6-week-old female BALB/c nude mice. After the average size of tumors reached 50 cm^3, we measure the growth of the tumors every two or three days. The treatment of each group is described below. All the mice were purchased from SPF Biotechnology (Beijing, China).

Coimmunoprecipitation (Co-IP)

The cells were lysed in IP lysis buffer (Thermo Scientific, USA) containing protease inhibitor cocktail. The cell lysates were incubated with anti-β-catenin antibody at 4 °C overnight, and immunoprecipitated with protein A+G-Sepharose beads (Thermo Scientific, USA) with shaking at 4 °C for 2 h. The beads were washed three times with IP lysis buffer. The immunoprecipitates were eluted and analyzed by western blotting.

Immunofluorescence

Cells were fixed to coverslips with 4% paraformaldehyde. β-catenin protein was then stained with its specific antibodies and fluorescent second antibodies. After that, the cells were stained with DAPI (4',6-diamidino-2-phenylindole). Finally, the slides were observed under a fluorescence microscope.

TOP/FOP flash reporter assay

The TOP/FOP-Flash reporter and pTK-RL plasmids were cotransfected into cells. After transfection for 48 h, a Dual - Luciferase Assay Kit (Promega) was utilized according to the

manufacturer's instructions to detect the activities of both the firefly and Renilla luciferase reporters. TOP - Flash and FOP-Flash reporter activity levels were calculated as the relative ratio of firefly luciferase activity to Renilla luciferase activity.

Dual-luciferase report assay

The WT-DKK1-3'UTR and MUT-DKK1-3'UTR reporter plasmids containing the human DKK1 3'-UTR sequence with wild-type or mutant-type miR-146a binding sites were constructed respectively. Then, the WT-DKK1 or MUT-DKK1 reporter were cotransfected with mimic-NC or miR-146a into CAL-62 cells with or without KDM1A knockdown. After transfection for 48 h, the cells were harvested to assess the luciferase activity using the Dual-Glo Luciferase system (Promega).

Transwell assay

In vitro cell migration assays were performed using polycarbonate membrane Transwell chamber (8 mm pore size; Corning, USA) in 24-well plates. RPMI 1640 medium (500 μL) containing 20% FBS was added to the lower chamber. The 150 μL cell suspension (1×10^6 cells/mL) deprived of serum was cultured in the upper chamber at 37 °C with 5% CO_2 for 24 h. The Transwell chamber was fixed in 5% glutaraldehyde and stained with 0.1% crystal violet. For the in vitro cell invasion assay, the Transwell chamber was coated with Matrigel (BD Biosciences, USA) before adding the cell suspension.

Assessment of apoptosis

The rates of apoptotic cells were measured by flow cytometry after staining with Annexin V/propidium iodide (BD Pharmingen, Franklin Lakes, USA). The data were analyzed using FlowJo software (version 10.0.7).

Additional file 2

1. Primers for RT-qPCR

Name	Forward Primer (5'-3')	Reverse Primer (5'-3')
β-actin	GCATGGGTCAGAAGGATTCCT	TCGTCCCAGTTGGTGACGAT
NANOG	ACCTATGCCTGTGATTTGTGG	AGTGGGTTGTTTGCCTTTGG
OCT4	TCCACTTTGTATAGCCGCTGG	TGCATACACACAAACACAGCAA
SOX2	TCAGGAGTTGTCAAGGCAGAG	CGCCGCCGATGATTGTTATTA
CD133	AGTCGGAAACTGGCAGATAGC	GGTAGTGTTGTACTGGGCCAAT
CD44	CTGCCGCTTTGCAGGTGTA	CATTGTGGGCAAGGTGCTATT
ALDH1	CCGTGGCGTACTATGGATGC	GCAGCAGACGATCTCTTTCGAT
KMT2F	CAGTGGCGGAACTACAAGCTC	CATAGCGGTACACCTTCTGAGA
KMT2A	TCTCCTCGCGGATCATTAAGA	GGTGTAGACTCTAATCGGGCAA
KMT7	TCACGGAGAAAAGAACGGACG	ATCATCCACATAATACCCCTCCA
KMT3C	GAATCCCTCGGAGACTGTAAGA	CGAAGGTGTTCTCTCTGGGT
KMT3E	TTCACCATCTGTAATGCGGAGA	ACACAATCGAACAGTTGGGGT
KMT2H	ACACTGTCCTTCAAAACGAGAC	GAAGAGTAGATGGCGTTGCATTA
KMT1A	CCTGCCCTCGGTATCTCTAAG	ATATCCACGCCATTTCACCAG
KMT1B	TCTATGACAACAAGGGAATCACG	GAGACACATTGCCGTATCGAG
KMT1C	TCCAATGACACATCTTCGCTG	CTGATGCGGTCAATCTTGGG
KMT1D	CATGCAGCCAGTAAAGATCCC	CTGCTGTCGTCCAAAGTCAG
KMT1E	TAAGACTTGGCACAAAGGCAC	TCCCCGACAGTAGACTCTTTC
KMT8	AATTTGGGATGGATGTGCATTG	GGCGCGATTGGCTTTAAAGT
KMT6B	GTCACTGAACACAGTTGCATTG	TGCACAAAACCGTCTCATCTTC
KMT6	AATCAGAGTACATGCGACTGAGA	GCTGTATCCTTCGCTGTTTCC
KMT3B	GAGCTACCTGTCCTTAGGAGAA	GACTCAGGATCATTTGTGCAGT
KMT3G	TTATTCCAGCCGACAAGCTGA	CGCAGTTTGGCATCGTGTG
KMT3F	CAGTACCCAGCTACAACAGAAG	CATTGGCTGACCCATTAGGATAC
KMT4	CTGCCGGTCTACGATAAACATC	AGCTTGAGATCCGGGATTTCT
KMT5A	ACAAATGCTCTGGAATGCGTT	CCGGCTAATGGTTTCCCCTG
KMT5B	GAGAAATGGAGGCAAGTTGTCT	ACATAGCGACTCTGTCCTTCA
KMT5C	CGCCTTCATCAACCATGACTG	GCCGTAGAAGCATGTCACC
KDM1A	TGACCGGATGACTTCTCAAGA	GTTGGAGAGTAGCCTCAAATGTC
KDM1B	CTCTCCTGTGGGGAACATTTC	GACTAGGTTCGGTTTTGCCATT

KDM5A	GTCTAAAGTGGGTAGTCGCTTG	GTTTGGGTATCAGTGCTGAGAA
KDM5B	AGTGGGCTCACATATCAGAGG	CAAACACCTTAGGCTGTCTCC
KDM5C	TCAGTGACAGTAAACGGCACC	ACACCGGCATCACATTTAGGT
KDM5D	CAAGACCCGCTTGGCTACATT	TTGGACGCGAGGAGTAAATCT
NO66	CGCTACATCAACGGACGAC	CAAAGCCCTGCGAGTTAGG
KDM3A	ACAGTGGCCTGCAATAACGTA	TCCCAGAAAGCGAACAGAAGT
KDM3C	TAACTCCTGCACCCCATTTACT	ATGAGCACTCTCTAGTCGTGG
KDM4A	ATCCCAGTGCTAGGATAATGACC	ACTCTTTTGGAGGAACAACCTTG
KDM4B	ACTTCAACAAATACGTGGCCTAC	CGATGTCATCATACGTCTGCC
KDM4C	CGAGGTGGAAAGTCCTCTGAA	GGGCTCCTTTAGACTCCATGTAT
KDM7C	CTCCCCTACGACGTTACCC	CAGTGGTATATGTCGATGTCGG
KDM6B	TTGGGCAACTGTACGAGTCAG	CCATAGTTCCGTTTGTGCTCAAG
KDM6A	GGACATGCTGTGTCACATCCT	CTCCTGTTGGTCTCATTTGGTG
KDM2B	GGGTTCCCCTGATATTTCGAGA	GCTCCCCACTAGGAGTTTGAC
CCND1	CGTGGGCTCTAAGATGAAGG	TGCGGATGATCTGTTTGTTC
C-MYC	GGCTCCTGGCAAAAGGTCA	CTGCGTAGTTGTGCTGATGT
CTNNB1	GCGCCATTTTAAGCCTCTCG	AAATACCCTCAGGGGAACAGG
DKK1	CCTTGAACTCGGTTCTCAATTCC	CAATGGTCTGGTACTTATTCCCG
APC2	CTGAAGCACCTACAGGGAAAA	CTGGAACTTGAGGTTGTACAGG
HIF-1α	CTCAAAGTCGGACAGCCTCA	CCCTGCAGTAGGTTTCTGCT
miR-146a	GACAGGGTCTCTCTCTGTG	CTAGCCTGGGCAACATGGA
U6	GCTTCGGCAGCACATATACTAAAAT	CGCTTCACGAATTTGCGTGTCAT

2. Sequences of siRNA and shRNA

Name	Sequence (5'-3')
shKDM1A-1	CACAAGGAAAGCTAGAAGA
shKDM1A-2	AACAATTAGAAGCACCTTA
siNC	UUCUCCGAACGUGUCACGUTT
siAPC2-1	CCUACAGGGAAAACUGGAGTT
siAPC2-2	CUGAAGAUGAUGAAAGUAAUU
siDKK1-1	TGATAGCCCTGTACAATGCTGCT
siDKK1-2	CGAUUUGGAGGUACCAUAAAGGAUU

3. Primers for ChIP-qPCR

Name	Forward Primer (5'-3')	Reverse Primer (5'-3')
APC2 P1	GAGCTTGCAGTGAGCCGAGAAC	GAAAAGATCCCCTGTGTCTGCCC
APC2 P2	AGACAGCCAGGCCGAAG	CTTCCCACCCTCTCCTATCATT
APC2 P3	CCCTTTCTGTGCTGTGAGTCTGTG	GGACACCTGGAGATGGCGACT
APC2 P4	GAGACAGAAAGAGAGAGGGACAT	TGGACCCGAACCCTGTC
APC2 P5	CCCCGCCAAGATGCTGATGTAAC	CAGACGCAGGAGGTTTTAAGAGGC
APC2 P6	ACACAGGAATCTTGGTCCTC	CTGTCTCACCCGCATCC
APC2 P7	AGCAGAGCCAGAAAGCAGAAACTG	CAAGGGAGGGCAGGCTCCAG
DKK1 P1	CCCACCACCCATCTCACCAA	CAATGCCAGGGCTCTAGGCT
DKK1 P2	CGTGGTCCCGTGCAAGGTAAG	CACATTAGCCCACCACTGAGAAGG
DKK1 P3	GACTGACCTGGCACGTTGGAC	CAAGTTCATCTACCGCCGCGATT
DKK1 P4	GGGCAACTGAAGGACCTCAAAGC	CAACTTGCACCCGCCACCTG
DKK1 P5	CAGGCAAGGGCACCCAAGTTC	GTCTGCTATAACGCTCGCTGGTAG
DKK1 P6	CCTCCCAGCGCTTTGAAAT	GTATAAAGGCAGCCGCGG
DKK1 P7	ACTGCATGCAAAGCAGACTTGT	TCACAAATCCTTCCTCATTCCACTCT
miR-146a	GACAGGGTCTCTCTCTGTG	CTAGCCTGGGCAACATGGA

4. Antibodies used in this study

Antibodies name	Application	Supplier	Cat #
Anti-human KDM1A antibody	WB, IHC	abcam	ab129195
Anti-human GAPDH antibody	WB	Cell Signaling Technology	5174
Anti-human OCT4 antibody	WB	Cell Signaling Technology	2750
Anti-human CD133 antibody	WB	Cell Signaling Technology	64326
Anti-human NANOG antibody	WB, IHC	Cell Signaling Technology	4903
Anti-human H3K4me1 antibody	WB, ChIP	Cell Signaling Technology	5326
Anti-human H3K4me2 antibody	WB	Cell Signaling Technology	9725

Anti-human H3K4me3 antibody	WB	Cell Signaling Technology	9751
Anti-human H3K9me1 antibody	WB	Cell Signaling Technology	14186
Anti-human H3K9me2 antibody	WB	Cell Signaling Technology	4658
Anti-human H3K9me3 antibody	WB	Cell Signaling Technology	13969
Anti-human H3 antibody	WB	Cell Signaling Technology	4499
Anti-human Ki67 antibody	IHC	Cell Signaling Technology	9449
Anti-human Cleaved caspase-3 antibody	IHC	Cell Signaling Technology	9661
Anti-human APC2 antibody	WB, IHC	abcam	ab113370
Anti-human DKK1 antibody	WB, IHC	abcam	ab109416
Anti-human CCND1 antibody	WB, IHC	Cell Signaling Technology	55506
Anti-human C-MYC antibody	WB, IHC	Cell Signaling Technology	18583
Anti-human β-catenin antibody	WB, IHC, IP, IF	Cell Signaling Technology	8480
Anti-human HIF-1α antibody	WB, IP, ChIP	Cell Signaling Technology	36169
Anti-human pan methyl Lysine antibody	WB	abcam	ab7315
Anti-human ubiquition antibody	WB	abcam	ab140601
Goat Anti-Rabbit IgG (HRP)	WB	abcam	ab6721
Normal Rabbit IgG	ChIP	Cell Signaling Technology	2729
Cy3-labeled Goat Anti-Rabbit IgG (H+L)	IF	Beyotime	A0516
